# Isolation and Functional Characterization of a Lycopene β-cyclase Gene Promoter from Citrus

**DOI:** 10.3389/fpls.2016.01367

**Published:** 2016-09-13

**Authors:** Suwen Lu, Yin Zhang, Xiongjie Zheng, Kaijie Zhu, Qiang Xu, Xiuxin Deng

**Affiliations:** Key Laboratory of Horticultural Plant Biology, Ministry of Education, Huazhong Agricultural UniversityWuhan, China

**Keywords:** citrus, carotenoid, lycopene β-cyclase, promoter, *cis*-element, enhancer

## Abstract

Lycopene β-cyclases are key enzymes located at the branch point of the carotenoid biosynthesis pathway. However, the transcriptional regulatory mechanisms of *LCYb1* in citrus with abundant carotenoid accumulation are still unclear. To understand the molecular basis of *CsLCYb1* expression, we isolated and functionally characterized the 5′ upstream sequences of *CsLCYb1* from citrus. The full-length *CsLCYb1* promoter and a series of its 5′ deletions were fused to the β-glucuronidase (*GUS*) reporter gene and transferred into different plants (tomato, *Arabidopsis* and citrus callus) to test the promoter activities. The results of all transgenic species showed that the 1584 bp upstream region from the translational start site displayed maximal promoter activity, and the minimal promoter containing 746 bp upstream sequences was sufficient for strong basal promoter activity. Furthermore, the *CsLCYb1* promoter activity was developmentally and tissue-specially regulated in transgenic *Arabidopsis*, and it was affected by multiple hormones and environmental cues in transgenic citrus callus under various treatments. Finer deletion analysis identified an enhancer element existing as a tandem repeat in the promoter region between -574 to -513 bp and conferring strong promoter activity. The copy numbers of the enhancer element differed among various citrus species, leading to the development of a derived simple sequence repeat marker to distinguish different species. In conclusion, this study elucidates the expression characteristics of the *LCYb1* promoter from citrus and further identifies a novel enhancer element required for the promoter activity. The characterized promoter fragment would be an ideal candidate for genetic engineering and seeking of upstream *trans*-acting elements.

## Introduction

Carotenoids are a class of 40-carbon terpenoid molecules present in most tissues of higher plants. They are one type of the most important secondary metabolites, and play a variety of roles in many biological processes in plants, including flowering and fruit coloration (Bartley and Scolnik, 1995), light harvesting for photosynthesis (Frank and Cogdell, 1996; Robert et al., 2004), protection from excessive light (Müller et al., 2001), defense against biotic and abiotic stresses (Ramel et al., 2012), and production of apocarotenoid hormones such as abscisic acid (ABA) and strigolactone (Creelman et al., 1987; Alder et al., 2012). As an important dietary component for human body, carotenoids provide precursors for vitamin A synthesis (Lakshman and Okoh, 1993; DellaPenna and Pogson, 2006) and reduce the risks of cardiovascular diseases, cancers and age-related diseases (Fraser and Bramley, 2004; Krinsky and Johnson, 2005; Botella-Pavía and Rodríguez-Concepción, 2006). Therefore, it is of great interest to understand the biosynthesis of these isoprenoids in plants.

Carotenoid metabolism is a complicated pathway involving the expression of many genes, which are regulated by various factors, such as developmental cues and environmental conditions (Liu et al., 2015; Nisar et al., 2015). Although a number of genes related to carotenoid synthesis and degradation have been isolated and analyzed, only a few studies have addressed the regulatory mechanisms of these processes. Recently, only three types of transcription factors, namely RAP2.2 (APETALA2/ethylene-responsive; Welsch et al., 2007), PIFs (phytochrome-interacting factors; Toledo-Ortiz et al., 2010) and RIN (ripening-inhibitor; Vrebalov et al., 2002; Fujisawa et al., 2013), have been identified to directly interact with the promoters of carotenogenic genes and regulate their expression. To gain insight into the complex regulatory mechanisms of this metabolic pathway and to unravel the underlying upstream interacting factors, functional characterization of the gene promoters is of great importance.

To date, some promoters of the genes in the carotenoid biosynthetic pathway have been analyzed and some progresses have been achieved. The analysis of the *Arabidopsis* phytoene synthase *(PSY)* promoter identified a G-box-like element involved in light induction and discrimination between different light qualities, and also identified a novel *cis-*acting element ATCTA which contributes to strong basal promoter activity (Welsch et al., 2003). The tomato phytoene desaturase *(Pds)* promoter developmentally drives high *GUS* expression in the organs where chromoplasts are formed, and promotes gene transcription in green tissues in response to end-product regulation (Corona et al., 1996). The *Arabidopsis* carotenoid cleavage dioxygenases 7 (*AtCCD7*) promoter exhibits a vascular-specific expression pattern in transgenic plants (Liang et al., 2011), while the β-carotene hydroxylase (*AtBCH*) promoter shows strong constitutive expression in dicot plants (Liang et al., 2009). The functional characterization of the *Gentiana lutea* zeaxanthin epoxidase (*GlZEP*) promoter in transgenic tomato plants showed that *GlZEP-GUS* expression is closely associated with fruit development and chromoplast differentiation, suggesting an evolutionarily conserved link between *ZEP* and the differentiation of organelles that store carotenoid pigments (Yang et al., 2012). Imai et al. (2013) isolated the promoter of the carotenoid cleavage dioxygenase 4a-5 gene of *Chrysanthemum morifolium* (CmCCD4a-5) and assessed its petal-specific promoter activity.

Lycopene β-cyclases are key enzymes functioning at the branch point of the carotenoid biosynthesis pathway and converting upstream red lycopene to downstream bright yellow α-/β-carotene (Cunningham et al., 1996). As an important economic fruit crop, citrus contains abundant carotenoids, and the carotenoid content and composition vary greatly among different species (Fanciullino et al., 2006; Xu C.-J. et al., 2006; Xu J. et al., 2006). Previous studies have reported that the carotenoid accumulation in citrus is closely related to the transcript levels of Lycopene β-cyclase genes (Kato et al., 2004). There are two types of lycopene β-cyclase genes (here designated as *LCYb1* and *LCYb2*, respectively) in citrus. *LCYb1* is predominantly expressed in leaf tissues, while *LCYb2* is mainly expressed in fruit tissues and shows a marked induction during fruit development (Alquézar et al., 2009; Mendes et al., 2011). It has been demonstrated that a relatively low transcript level of *LCYb2* (also named as β-*LCY2 / LCYB2*) results in lycopene accumulation in red grapefruit (*Citrus paradisi*) (Alquézar et al., 2009; Mendes et al., 2011; Alquezar et al., 2013). However, studies on ‘HongAnliu’ sweet orange (a red-flesh mutant of ‘Anliu,’ *C. sinensis*) revealed that the down-regulation of both *LCYb* (*LCYb1*) and *capsanthin capsorubin synthase (CCS)* (*LCYb2*) may be responsible for the abnormal lycopene accumulation in the mutant (Xu et al., 2009; Yu et al., 2012). Zhang et al. (2012b) further elucidated that only *CitLCYb1* participates in the formation of α-carotene during the green stage in the flavedo, and that the high expression levels of both *CitLCYb1* and *CitLCYb2* during the orange stage play an important role in the accumulation of β, β-xanthophylls in citrus fruits. Apart from the roles in fruit color development, the high expression levels of *LCYb1* in leaf tissues suggest that this gene also participates in photosynthesis and other biological processes, which are crucial to the survival of plants. Additionally, since *LCYb1* is nearly present in all plant species and is an evolutionarily ancient and conserved gene, study of citrus *LCYb1* promoter will not only help us to understand the transcriptional regulatory mechanism of *LCYb1* in citrus, but also promote the understanding of *LCYb1* in other species. Although the promoters of *LCYb2* have been isolated and functionally analyzed in tomato (Dalal et al., 2010) and watermelon (Bang et al., 2014), little information is available regarding the *LCYb1* promoter.

The objectives of the present study were to isolate and functionally characterize the *CsLCYb1* promoter from sweet orange (*C. sinensis*) as well as to analyze the *LCYb1* promoters from different citrus species. This study will contribute to understanding the expression characteristics of *LCYb1* promoters and is expected to help future transcriptional regulation studies of *LCYb1* expression in citrus.

## Materials and Methods

### Plant Materials

The materials included four genotypes of pummelo (*C. grandis*; White-flesh Guanxi pummelo, Red-flesh Guanxi pummelo, Huanong red pummelo, HB pummelo), three genotypes of grapefruit (*C. paradisi*; Star Ruby grapefruit, Marsh grapefruit, and Flame grapefruit), four genotypes of sweet orange (*C. sinensis*; Washington navel orange, Cara Cara navel orange, Anliu sweet orange, HongAnliu sweet orange), and three genotypes of mandarin (*C. reticulata*; Bendizao mandarin, Qingjiang ponkan, Mangshan wild tangerine). Leaves of all these citrus varieties were obtained from the National Center of Citrus Breeding, Huazhong Agricultural University, Wuhan, China. The tissues were frozen in liquid nitrogen and stored at -80°C until use. Tomato (*Lycopersicon esculentum cv* Ailsa Craig) and *Arabidopsis* (*Arabidopsis thaliana*, ecotype Col-0) plants were grown under standard greenhouse conditions. Embryogenic callus used in this study was derived from Marsh grapefruit and subcultured on solid MT (Murashige and Tucker) basal medium containing 50 g L^-1^ sucrose under normal conditions (16 h light/8 h dark cycles at 25°C).

### Promoter Cloning and Sequence Analysis

The *CsLCYb1* cDNA sequence (orange1.1t00772) was used as a query to search the *C. sinensis* genomic database^1^ (Xu et al., 2013) and the 5′ upstream genomic sequence (about 2 kb) was retrieved (chrUn:9346020..9348020). Specific primers for promoter isolation were designed based on the reference sequence (Supplementary Table S1). Briefly, genomic DNA was extracted from leaves of Anliu sweet orange, White-flesh Guanxi pummelo, Marsh grapefruit and Bendizao mandarin using the CTAB (cetyltrimethylammonium bromide) method (Cheng et al., 2003). PCR reactions were performed under the following conditions: 95°C for 3 min, followed by 32 cycles at 95°C for 10 s, 55°C for 20 s and 72°C for 1 min, and a final 7 min extension at 72°C. The PCR products were gel-purified and cloned into the pMD18-T vector (TaKaRa, Dalian, China) for sequencing. The first nucleotide acid of the *CsLCYb1* mRNA was set as the transcription start site (TSS). Promoter regions and plant regulatory motifs were searched using the Softberry TSSP and Nsite-PL program^2^. A search for putative *cis*-elements in the promoter sequence was performed by using the PLACE^3^ (Higo et al., 1999) and PlantCARE^4^ (Lescot et al., 2002) databases. The *LCYb1* promoter in mandarin was retrieved from the Citrus clementina genome database ^5^. Multiple sequence alignments were performed using the ClustalX2 and GeneDoc programs.

### Vector Construction

The entire *CsLCYb1* promoter region (-1584 bp from the ATG start codon) and its five deletions (gradually truncated from the 5′ end of the *CsLCYb1* promoter) were amplified by PCR from the pMD18-T basic vector containing the 5′ full-length flanking sequence. Specific primers with EcoRI and NcoI restriction sites were designed (Supplementary Table S1). The amplified fragments were double digested and inserted into the corresponding site of the plasmid pCAMBIA1301 (CAMBIA, Canberra, Australia) in the upstream of the β-glucuronidase (GUS) reporter gene, replacing the *CaMV35S* promoter (the cauliflower mosaic virus 35S promoter). The six vectors were designated as LP (-1584), LP1 (-1255), LP2 (-1045), LP3 (-746), LP4 (-406), and LP5 (-247), respectively. The CaMV35S::GUS fusion in pCAMBIA1301 was used as a positive expression control (designated as 35S).

Internal deletion vector construction: Double stranded DNA, (GTGACTGAAATCATCAACCCTTGATGAACATCCTTTGCTATTGGGCATGAATGGAGAAGGAAGAAAATGAG (ATTGAAGGAAGAAAAATGAG)_n_ CGTGAAGGAGGAAAAGTGAGAAGAAAAAAAATTATATATTTTTTAATT), was synthesized to yield two fragments denoted as W1 (*n* = 1) and W2 (*n* = 2), respectively. Then, two pairs of primers (LMa-F and LMa-R; LMb-F and LMb-R) were used for PCR amplification of the full-length *CsLCYb1* promoter sequences to obtain two fragments denoted as LMa and LMb, respectively. Next, overlapping PCR was performed with three fragments (LMa, LMb, W1 or W2) simultaneously as templates and with two oligonucleotides (LPF and LPR containing EcoRI and NcoI sites at their 5′-ends) as primers. The obtained fragments were double digested and subcloned into the correspondingly enzymatic sites of the pCAMBIA1301 plasmid to yield two internal deletion vectors WP1 and WP2. The promoter-*GUS* vectors are schematically represented in **Figures 2** and **6**. All constructs were verified by sequencing and then transformed into the *Agrobacterium tumefaciens* stain GV3101 by the freeze-thaw method. The generated constructs were subsequently transformed into plants to test promoter activities.

### Plant Transformation

Tomato transient transformation was performed according to the method described by Orzaez et al. (2006) with minor modification. *Agrobacterium* cultures (0.5 mL) from individual colony were grown at 28°C for 24 h in LB liquid medium supplemented with kanamycin (100 mg L^-1^) and rifampicin (25 mg L^-1^), then transferred to 50 mL induction medium (LB medium plus 20 mM acetosyringone, 10 mM MES, pH 5.6) containing corresponding antibiotics and grown again. In the following day, the bacterial cells were sedimented by centrifugation and re-suspended in infiltration medium (10 mM MgCl_2_, 10 mM MES, 20 mM acetosyringone, pH 5.6) to an OD600 of approximately 1.0, and then incubated at room temperature with gentle agitation (20 rpm) for about 2 h. Cultures were collected with a syringe, and then injected into detached tomato fruits (*L. esculentum* cv Ailsa Craig) at a total volume of 600 μl. Three days later, the injected fruits were cut into slices for histochemical GUS staining.

*Arabidopsis* transformation was done using the floral dip method established by Clough and Bent (1998). Two generations of the transformed plant were selected on MS (Murashige and Skoog) medium supplemented with 25 mg l^-1^ of hygromycin, then were transferred to soil, and finally were grown in the greenhouse at 22°C under a 16 h light/8 h dark photoperiod. The positive transformations were further confirmed through PCR amplification of genomic DNA by using the primer sets, respectively (Supplementary Table S1). The estimation of transgene copy numbers in transgenic *Arabidopsis* was conducted according to the method described by Weng et al. (2004). The results are shown in Supplementary Figure S3. Finally, approximately 10 independent homozygous T_2_ transgenic lines with single-copy insertion of each promoter were used for subsequent GUS assays, and the wild type *Arabidopsis* plants were used as the negative control. Different tissues, including roots, stems, leaves, flowers, and fruits, were collected from each selected plant growing in soil for about 45 days for tissue-specific expression assay. Seedlings of the transgenic lines containing the full-length promoter construct (LP) were collected during five developmental stages for developmental expression assay. Seedlings of other transgenic lines were only collected on day 24 after seed germination to compare the promoter activities among different truncated fragments.

Embryogenic callus transformation was performed with the method described by Li et al. (2002). Positive transgenic lines were screened and subcultured with the method described by Cao et al. (2012). Transgenic callus was selected on solid MT (Murashige and Tucker) basal medium containing 50 mg l^-1^ of hygromycin and 250 mg L^-1^ of cefotaxim. PCR amplification was used to further confirm the positive lines. Each independent line was propagated on solid MT basal medium containing 50 g L^-1^ sucrose under normal conditions (16 h light/8 h dark cycles at 25°C). Twenty-day-old callus was harvested for GUS assays or various stimuli treatments.

### Stress Treatments

In various stimuli treatments, transgenic callus was cultured on solid MT medium for about 20 days at 25°C, followed by culturing for 4 days in liquid MT medium with shaking. The stable cell suspension cultures in a good state were immersed in MT liquid medium supplemented with 100 μM ABA, 100 μM auxin (IAA), 100 μM gibberellin (GA), 100 μM salicylic acid (SA), 100 μM Methyl Jasmonate (JA), 100 μM Kinetin (KT), 10% (w/v) sucrose (Suc), 10% (w/v) glucose (Glu), 200 mM NaCl for 12 h under light. Callus cells in MT liquid medium without any supplement were used as negative control. All parallel samples were grown under the same conditions. After treatment, all samples were frozen in liquid nitrogen and used for subsequent GUS assays. Three biological replicates were performed for each set of treatments.

### GUS Assays

Histochemical staining and fluorometric assays were performed according to the method proposed by Jefferson et al. (1987).

Various tissues were submerged in X-gluc buffer [100 mM phosphate buffer (pH 7.0), 1 mM 5-bromo-4-chloro-3-indolyl-glucuronide (X-gluc) solution, 0.1% Triton X-100, 10 mM EDTA, 0.5 mM potassium ferrocyanide, 0.5 mM potassium ferricyanide, and 20% methanol] overnight at 37°C. After staining, the tissues were kept in 70% ethanol until the chlorophyll was removed, and then photographed with a digital camera or under a stereomicroscope (Leica MZFL III). All the experiments were repeated ten times for each construct.

Quantitative GUS assays were performed as follows. The samples were frozen in liquid nitrogen and ground into powder. Protein was extracted with GUS extraction buffer (50 mM phosphate buffer, pH 7.0; 10 mM EDTA; 0.1% Triton X-100; 0.1% Sodium Dodecyl Sulfate; and 10 mM β-mercaptoethanol). After centrifugation, the supernatant of various extraction liquids was used for the subsequent protein quantification and fluorometric assays. Protein concentrations were determined using the BCA Protein Assay Kit (Beyotime Biotechnology, China). Fluorometric assays were performed in microtiter plates at 37°C in the presence of 1 mM 4-methylumbelliferyl glucuronide (MUG, Sigma–Aldrich). The appearance of 4-methylumbelliferone (MU) was monitored using a Tecan Infinite^TM^ M200 plate reader at 365 nm excitation and 455 nm emission. GUS enzyme activity was expressed as umoles of 4-MU per min per mg protein. Three replicates were performed for each sample.

### Simple Sequence Repeat (SSR) Screening

Total genomic DNA was extracted from leaf samples of different citrus varieties. SSR screening primers (LSSR-F and LSSR-R) were designed according to above isolated *LCYb1* promoter sequences (Supplementary Table S1). The SSR amplification reactions were conducted according to the protocol described by Chai et al. (2013). The amplification products were firstly checked by agarose gel electrophoresis. Then, PCR products were separated by polyacrylamide gel electrophoresis and visualized by silver staining following the protocol developed by Ruiz et al. (2000).

### Statistical Analysis

The data were presented as mean ± SD of three independent experiments. Statistical analyses were done using the One-way ANOVA test on the Microsoft Excel program (Microsoft Office, 2010). The difference with a *P*-value <0.05 (^∗^/*Lowercase letters*) or <0.01(^∗∗^/*Uppercase letters*) was considered as significant.

## Results

### Isolation and Sequence Analysis of the *CsLCYb1* Promoter

**FIGURE 1:**
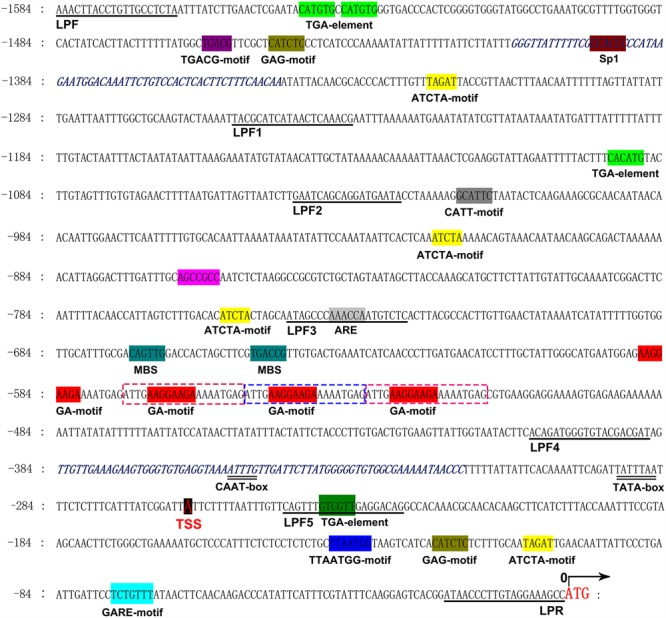
**The 5′ upstream promoter sequences of the *CsLCYb1* gene.** Numbers indicate the positions relative to the ATG start codon (0). The putative TATA-box (double underlined), CAAT-box (double underlined), transcriptional start site (TSS, highlighted), and some *cis*-elements (highlighted) are labeled under the sequences. Primers for amplifying a series of 5′ truncated fragments are also underlined and labeled. The pair of reverse complementary sequences are italic in blue color. The 20 bp tandem repeat sequences are dot outlined.

The *CsLCYb1* genomic DNA sequence and its 5′ flanking region were downloaded from the genomic database of sweet orange with the full length *CsLCYb1* cDNA (orange1.1t00772) as a query sequence. The 1584 bp fragment located in the upstream from the ATG start codon was obtained through PCR-based method and tentatively designated as the full-length promoter of *CsLCYb1* (**Figure 1**). The TSS was located at -263 bp upstream from the ATG (the position of the ATG start codon was designated as 0). Bioinformatics analysis revealed that the *CsLCYb1* promoter was a typical eukaryotic promoter containing a potential TATA box at -292 bp, a CAAT-box at -356 bp, and many TA-rich enhancer elements. A large number of hormone-responsive elements were predicted in the promoter, such as the ATCTA-motif in response to ethylene, the CGTCA-motif to Jasmonate, the GARE motif to gibberellin, the TCA motif to SA and the TGA motif to auxin. We also discovered some stress-responsive elements, such as the ARE-motif involved in anaerobic induction, the CATGTG-motif in dehydration response, the E-box in defense signaling, and the MYB-binding sites in drought inducibility. In addition, the *CsLCYb1* promoter carried numerous light-responsive elements, such as the Box 4, Box II, CATT-motif, GA-motif, GAG-motif, SP1 and TCCC-motif. Among these motifs, the GA-motif characterized by the AGATT sequence existed as a tandem repeat in the promoter region between -588 and -522 bp. We also compared the *cis*-elements in the *CsLCYb1* promoter with those in the previously isolated *CsPSY* promoter (Zeng et al., 2013) and *CitCRISO* promoter (Eun et al., 2015) (Accession No. KJ751507) in citrus. Many common *cis*-acting elements were discovered, such as the CGTCA-motif that is involved in the MeJA-responsiveness and the SP1 element that responds to light. Some of the relevant *cis-*elements and their relative positions in the upstream of the ATG start codon are listed in Supplementary Table S2. Notably, a pair of reverse complementary sequences that were not completely symmetrical were discovered in the regions from -1409 to -1348 bp and from -384 to -318 bp.

**FIGURE 2:**
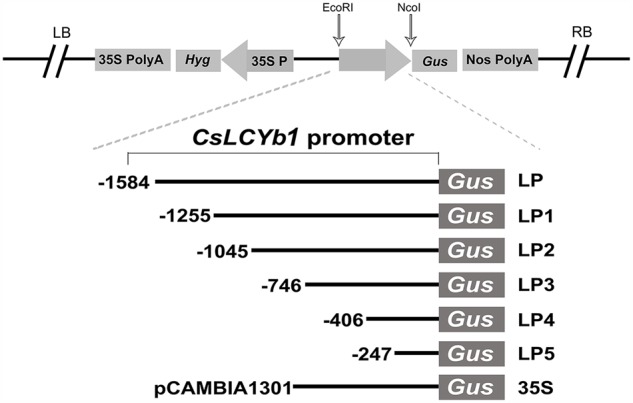
**Schematic representation of the *CsLCYb1* promoter::*GUS* vectors construction.** These constructs are based on the pCAMBIA1301 vector. LB, left border; 35S PolyA, Cauliflower Mosaic Virus 35S terminator; *Hyg*, hygromycin resistance gene; 35S P, Cauliflower Mosaic Virus 35S promoter; *GUS*, β-glucuronidase reporter gene; Nos PolyA, nopaline synthase terminator; RB, right border. Hollow arrows indicate the positions of the promoter insertion in the vectors. The promoters contain the full-length sequence (LP) and its five 5′ truncated fragments (LP1, LP2, LP3, LP4, and LP5). Numbers indicate the sequence length from the first base of the ATG.

### Transient Expression Assay of *CsLCYb1* Promoter in Tomato

**FIGURE 3:**
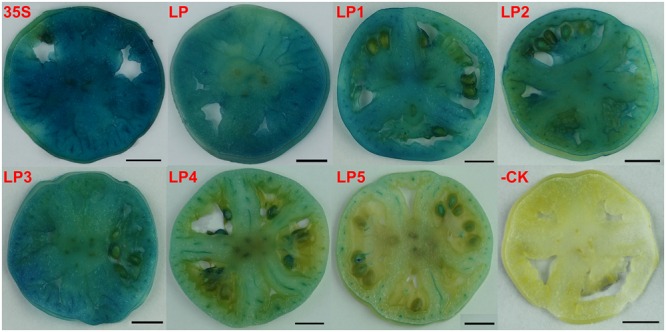
**Histochemical GUS staining of tomato green fruit.** Transgenic lines carrying the *GUS* reporter gene under the control of the CaMV35S promoter were used as the positive control (35S) and untransformed tomato was used as the negative control (-CK). LP, LP1, LP2, LP3, LP4, and LP5 represent transgenic lines under the control of the full-length *CsLCYb1* promoter and its five 5′ truncated fragments, respectively. Bars, 1 cm.

Firstly, we applied a transient expression method to identify whether the cloned *CsLCYb1* promoter sequence was active. Tomato fruits at the mature green stages were injected with bacterial cultures carrying each promoter::*GUS* construct, respectively. Fruits were harvested 3 days later and transverse sections were stained for *GUS* expression. As expected, we found strong *GUS* staining in the fruits transformed with the 35S construct, while no *GUS* expression was detected in the wild type without transformation. GUS staining of the full-length promoter construct LP and the truncated promoter constructs LP1, LP2, and LP3 was evident in columella and placental tissues but not in seeds. The intensity of GUS staining was similar among LP, LP1, and LP3, while LP2 showed relatively lower GUS intensity compared with the above three constructs. However, in transgenic tomato LP4 and LP5, only the vascular bundles were stained (**Figure 3**).

### Spatial and Temporal Expression Patterns of *CsLCYb1* Promoter in *Arabidopsis*

**FIGURE 4:**
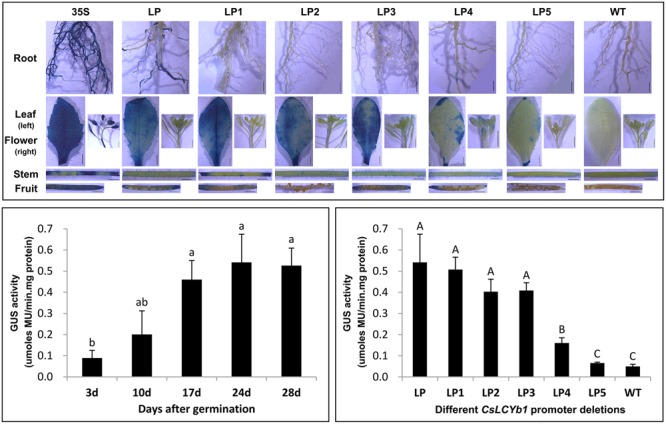
**GUS assays of transgenic *Arabidopsis* plants. (Up)** Qualitative GUS staining. Tissues (root, leave, flower, stem, and fruit) from each construct were separately subjected to histochemical GUS staining. Transgenic lines carrying the *GUS* reporter gene under the control of the *CaMV35S* promoter were used as the positive control (35S) and untransformed *Arabidopsis* were used as the negative control (WT). Bars, 2 mm. **(Down)** Quantitative GUS assays of transgenic *Arabidopsis* seedlings carrying the full-length promoter construct (LP) during seedling development (*at bottom left*) and transgenic *Arabidopsis* seedlings carrying different promoter constructs (*at bottom right*). Leaves were harvested on day 24 after seeding. Data are means ± SD of three independent experiments. *Lowercase letters* indicate significant differences at *P* < 0.05. *Uppercase letters* indicate significant differences at *P* < 0.01.

To elucidate the spatial and temporal expression patterns of the *CsLCYb1* promoter, we examined stable *Arabidopsis* transgenic plants. Homozygous single-insertion T_2_ lines of each construct were used for histochemical staining and quantitative GUS assays. Different tissues (roots, stems, leaves, flowers, and fruits) from each construct were subjected to histochemical GUS staining (**Figure 4**). GUS staining was observed in all tested tissues of the CaMV35S construct, but not in the wild type control. *GUS* expression of LP, LP1, LP2, and LP3 constructs was apparently detectable in leaves. No significant difference in GUS staining intensity was found among LP, LP1 and LP3, while the staining intensity of LP2 was relatively lower than that of the above-mentioned three constructs. Little or no GUS staining was found in the leaves of LP4 and LP5. In addition, several tissues of some constructs were slightly stained, such as the root of LP, flower of LP4, stem ends of LP1 and fruit ends of LP, LP1, LP3, and LP4. Epidermal hairs in the stems of LP, LP1, LP2, LP3, and LP4 were also stained.

Glucuronidase enzyme activities were quantified by fluorometric 4-MUG assay at different developmental stages of seedling in the full length promoter transgenic lines (**Figure 4**). The results showed that the promoter activities increased along with the seedling development, reached the maximum on day 24, and subsequently decreased on day 28. Then, *GUS* expression of different promoter constructs was compared on day 24. In accordance with the results of GUS staining assay, the LP construct carrying the full-length sequence of the *CsLCYb1* promoter produced the highest level of *GUS* expression in leaf tissues. With deletions of the 5′ fragments, the promoter activity gradually decreased. However, no significant difference in *GUS* activity was found among LP, LP1, LP2, and LP3. By comparison, the GUS activities of LP4 and LP5 were remarkably reduced. The GUS activity of LP4 was about fourfold lower than that of LP, and that of LP5 was too low to be detected.

### Responses of Different Promoter Constructs to Various Stimuli in Citrus Callus

**FIGURE 5:**
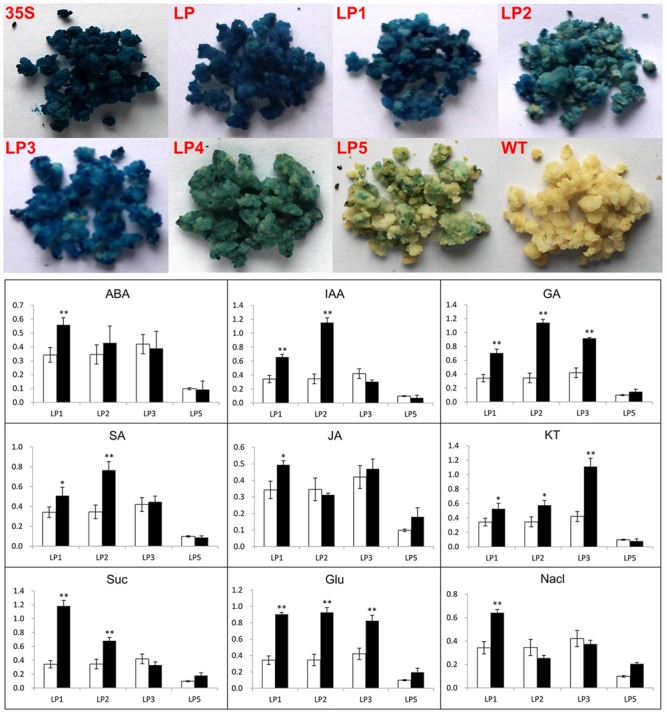
**GUS assays of transgenic citrus callus. (Up)** Qualitative GUS staining. Citrus callus carrying different promoter constructs were separately subjected to histochemical GUS staining. Transgenic lines carrying the *GUS* reporter gene under the control of the *CaMV35S* promoter were used as the positive control (35S) and untransformed callus was used as the negative control (WT). **(Down)** Quantitative GUS assays of different promoter deletions in stably transformed citrus callus under various treatments, including abscisic acid (ABA), auxin (IAA), gibberellin (GA), salicylic acid (SA), Methyl Jasmonate (JA), Kinetin (KT), sucrose (Suc), glucose (Glu), and NaCl. Data are means ± SD of three independent experiments. Significant differences between values are indicated by asterisk (^∗^*P* < 0.05, ^∗∗^*P* < 0.01).

Previously we detected the transcripts of *CsLCYb1* in citrus callus (data not shown). In order to eliminate the possible effect of heterogeneous expression on promoter activity, we stably transformed the promoter constructs into citrus callus, respectively. The expression level of each construct in transgenic citrus callus was also evaluated by histochemical GUS staining (**Figure 5**). The results showed that GUS staining was obviously visible in callus transformed with constructs LP, LP1, LP2, and LP3, suggesting that these fragments could functionally drive GUS expression in callus. The callus transformed with construct LP4 was slightly stained, indicating possible promoter activity of this fragment in callus. Pale blue was observed only in a few cells of transgenic callus with LP5.

Although the roles of phytohormones, glucose (sucrose or mannitol) and various other stimuli in carotenoid accumulation have been studied, little has been known about the molecular mechanisms that regulate carotenoid metabolism and gene expression. To fully reveal the regulation, we analyzed *CsLCYb1* promoter function in detail by *GUS* expression assay under various stimuli treatments. Based on the GUS staining results, we speculated that the sequence region from LP1 (-1255) to LP4 (-406) was likely to significantly contribute to the regulation of the *CsLCYb1* promoter activity. Thereby, we tested the expression levels of constructs LP1, LP2, and LP3 under various stimuli including hormones, sugar and salt stress to examine the responses of different 5′ sequence regions. *GUS* expression level driven by LP5 promoter was also tested under all conditions. As shown in **Figure 5**, LP1 promoter activity was significantly induced under ABA and JA treatments. In contrast, the promoter activities of LP2 and LP3 were not strongly affected by both ABA and JA. IAA treatment induced the activities of LP1 and LP2 promoter (1.9- and 3.3-fold, respectively), and SA treatment also induced their activities (1.5- and 2.2-fold, respectively). However, the LP3 construct did not show any significant differences in promoter activity under IAA and SA treatments. When callus cells were incubated with GA, we observed significant induction of *GUS* expression. Compared with under normal conditions, the promoter activities of LP1, LP2, and LP3 were increased to 2.1-, 3.3-, and 2.2-fold, respectively. Under KT treatment, *GUS* expression driven by either LP1 or LP2 was increased to about 1.6-fold. Notably, a deletion from LP2 to LP3 resulted in a sharp increase of *GUS* activity to 1.1 μmol MU min^-1^ mg^-1^ protein (2.6-fold). Both sucrose and glucose treatments significantly enhanced the promoter activities of LP1 and LP2. However, *GUS* expression of LP3 was only promoted by glucose, not by sucrose. Additionally, we analyzed the promoter activities under NaCl treatment to determine whether the promoter activity responded to salt stress. The results showed that the promoter activity of LP1 was significantly increased, while the activities of LP2 and LP3 were decreased to some extent, even though no significant differences were observed.

### Finer Deletion Analysis of the *CsLCYb1* Promoter

**FIGURE 6:**
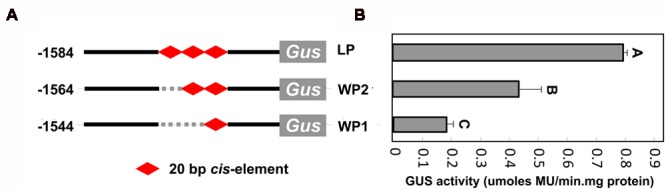
**Finer deletion analysis of the 20 bp fragment. (A)** Schematic representation of the internal deletion promoter constructs. Numbers indicate the sequence length from the first base of the ATG. **(B)** Quantitative GUS assays of different constructs in stably transformed citrus callus.

Since a deletion from LP3 to LP4 resulted in a significant reduction in promoter activity, we speculated that an enhancer (or enhancers) may be located in this region. Further sequence analysis revealed the existence of a 20 bp fragment (ATTGAAGGAAGAAAAATGAG) in the region as a tandem repeat (between -574 and -513 bp upstream from the ATG). A search of the PLACE database for the potential *cis*-elements in the 20 bp sequence identified five reported *cis*-elements: Inr-element (YTCANTYY), CAAT-box (CAAT), GT1-motif (GAAAAA), GT-element (GRWAAW), and pollen-specific element (AGAAA). In order to verify whether the 20 bp fragment was essential for promoter function, we performed finer deletion analysis. Additional vectors with the deletion of one or two copies of the 20 bp fragment were constructed and transformed into citrus callus to test the promoter activities. Compared with the complete *CsLCYb1* promoter, the deletion of one copy caused the promoter activity to dramatically decrease to 55%, while the promoter activity with the deletion of two copies dropped to approximately 23% (**Figure 6**). Taken together, these data clearly indicated that the 20 bp fragment acted as a positive *cis*-acting regulatory element to affect promoter activity.

### Sequence Analysis of *LCYb1* Promoters from Other Citrus Species

**FIGURE 7:**
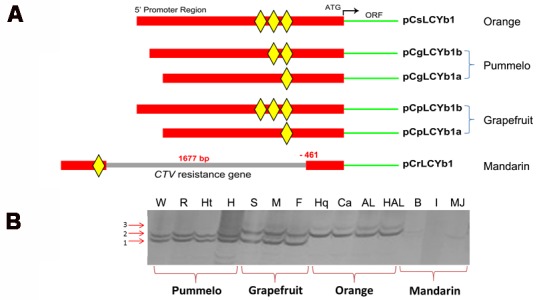
**Analysis of *LCYb1* promoters from various citrus species. (A)** Schematic representation of promoter structure of *LCYb1* from four citrus species. Green lines represent the coding sequences of *LCYb1* genes. Red lines represent the promoter sequences of *LCYb1*. Gray lines represent the inserted large fragment. The inserted position and fragment size are indicated. Yellow rhombuses represent the 20 bp enhancer elements. **(B)** SSR screening of different *LCYb1* promoters from various citrus varieties. Numbers on the left denote the three electrophoretic bands. Four citrus species including pummelo, grapefruit, sweet orange and mandarin were detected. W, White-flesh Guanxi pummelo; R, Red-flesh Guanxi pummelo; Ht, Huanong red pummelo; H, HB pummelo; S, Star Ruby grapefruit; M, Marsh grapefruit; F, Flame grapefruit; Hq, Washington navel orange; Ca, Cara Cara navel orange; AL, Anliu sweet orange; HAL, HongAnliu sweet orange; B, Bendizao mandarin; I, Qingjiang ponkan; MJ, Mangshan wild tangerine.

In order to further understand the sequence characteristics of *LCYb1* promoter, we isolated promoters of *LCYb1* alleles from other citrus species. Due to the high heterozygosity in citrus genome, most of the gene loci have two different alleles termed as a and b, respectively. *CsLCYb1* in sweet orange has two different coding sequences (data not shown), while we got only one promoter sequence named as pCsLCYb1. We tried our best but failed to get the other one. However, in pummelo and grapefruit, we obtained two different promoter sequences named as pCgLCYb1a and pCgLCYb1b, pCpLCYb1a and pCpLCYb1b, respectively (Supplementary Figure S1). Multiple sequence alignment revealed that these promoter sequences differed in the copy numbers of the 20 bp enhancer element in addition to the differences in several single nucleotide polymorphisms (SNPs; Supplementary Figure S2). We retrieved the *LCYb1* promoter in mandarin (named as pCrLCYb1) from the *C. clementina* genome databases and found that a large fragment was inserted in the enhancer region of the *LCYb1* promoter (Supplementary Figure S1). Interestingly, partial sequences of the large fragment were reversely complementary to a citrus tristeza virus (CTV) resistance gene according to the NCBI blast search, and the insertion resulted in the remaining of only one copy number of the 20 bp enhancer element in the promoter. The sequence characteristics of *LCYb1* promoters from four citrus clades are schematically represented in **Figure 7A**.

To further confirm the association between the copy numbers of the 20 bp enhancer element and genetic evolution of citrus species, a pair of primers was designed to develop a derived SSR (simple sequence polymorphism) DNA molecular marker (Supplementary Table S1). The primers LSSR-F and LSSR-R were used to amplify the promoter enhancer regions from four clades of citrus species (pummelo, mandarin, orange and grapefruit). Through the polyacrylamide gel electrophoresis method, three electrophoretic bands were separated clearly as shown in **Figure 7B**. According to the corresponding copy numbers, we defined these three bands as 1, 2, and 3. Pummelo had bands 1 and 2, while grapefruit had bands 1 and 3. Sweet orange only contained one band (3), while no band was found for mandarin. These results indicated that the SSR markers based on the 20 bp enhancer element could be used to distinguish different citrus species.

## Discussion

Lycopene β-cyclases are key enzymes catalyzing the cyclization of the linear *trans*-lycopene to produce the cyclic α- and β-carotenes in the carotenoid biosynthetic pathway (Cunningham et al., 1996). There are two Lycopene β-cyclase genes (*LCYb1* and *LCYb2*) in citrus. Functional analysis showed that both enzymes participate in the formation of β-carotene, and play important roles in fruit ripening and plant development (Alquézar et al., 2009; Xu et al., 2009; Mendes et al., 2011; Yu et al., 2012; Zhang et al., 2012b, 2013). Although the chromoplast-specific *LCYb2* is mainly responsible for the carotenogenesis in fruits, the role of *LCYb1* in leaf tissues and flavedo at green stage cannot be neglected. To elucidate the molecular basis of *LCYb1* gene expression and investigate the upstream interacting factors, we cloned the *LCYb1* promoter from citrus and analyzed its characteristics in detail for the first time. Even though *CsLCYb1* was found to be highly expressed in leaf tissues, its expression was also detected in other tissues including unripe fruit and citrus callus, as demonstrated by previous studies (Xu et al., 2009; Gao et al., 2011; Cao et al., 2012; Yu et al., 2012; Zhang et al., 2012b). We also previously detected the transcripts of *CsLCYb1* in these tissues (data not shown). Due to the difficulty of transformation, long life cycle (at least 1 year from sowing to fruit ripening), and the difficulty of GUS measurement of citrus species, we investigated the promoter activities by transient expression assay in tomato fruit and stable transformation method in *Arabidopsis* plants. These two methods had been successfully used for studying the promoter function of carotenogenic genes, such as the tomato phytoene desaturase (*SlPDS*; Corona et al., 1996) and chromoplast-specific Lycopene β-cyclase (*SlCYC-B*; Dalal et al., 2010), the *C. morifolium* carotenoid cleavage dioxygenase 4a-5 gene (*CmCCD4a-5*; Imai et al., 2013), the *Crocus sativu* carotenoid cleavage dioxygenase (*CsCCD*; Ahrazem et al., 2010), the *Arabidopsis* carotenoid cleavage dioxygenase (*AtCCD7*; Liang et al., 2011), the *C. unshiu* carotenoid isomerase (*CuCRTISO*; Eun et al., 2015), and the *G. lutea* zeaxanthin epoxidase (*GlZEP*; Yang et al., 2012). Tomato transient assay is an efficient and simple way, while *Arabidopsis* stable transformation is appropriate for temporal and tissue-specific detection. Further promoter detection in transgenic citrus callus can exclude the effect of heterogeneous background on promoter activity. Therefore, it is reasonable and appropriate to analyze *CsLCYb1* promoter function simultaneously in transgenic tomato green fruit, *Arabidopsis* plants and citrus callus.

### Expression Characteristics of Different *CsLCYb1* Promoter Deletions

Promoter deletion analyses performed, respectively, in tomato, *Arabidopsis* and citrus callus produced similar results (**Figures 3–5**), suggesting that the three analyzed species shared some transcription factors binding to the promoter. A similar finding was reported based on the functional analysis of a *dahlia mosaic virus* subgenomic transcript (DaMVSgt) promoter in transient protoplasts, transgenic tobacco and *Arabidopsis* plants (Banerjee et al., 2015). The 1584 bp upstream region from the translation start site displayed the maximum promoter activity, and the minimal promoter LP3 containing 746 bp upstream sequences was sufficient to drive strong *GUS* gene expression. Therefore, the minimal promoter LP3 could be a useful tool in genetic engineering. The truncated fragment LP4 containing core promoter element (TATA-box, CAAT-box, and TSS) exhibited very weak promoter activity (**Figures 1** and **5**). However, the shortest fragment LP5, which did not contain any core promoter element, also drove very little *GUS* expression in transgenic callus (**Figure 5**). One explanation may be that the actual positions of core elements in the promoter regions are not consistent with the bioinformatics predictions, which needs to be verified by more experiments. Another reason could be that the promoter sequences containing no core elements such as TATA-box still have promoter activity as demonstrated previously (Burke and Kadonaga, 1996; Nakamura et al., 2002). In the promoter expression assays, the GUS staining intensity of LP2 was slightly lower than that of LP1 and LP3 in tomato and *Arabidopsis*; however, this minor difference did not reach significant level as revealed by the quantitative results (**Figures 3** and **4**).

### Expression Patterns of *CsLCYb1* Promoter in Response to Various Exogenous and Endogenous Factors

Previous studies reported that the *CsLCYb1* transcripts accumulate predominantly in leaf and fruit flavedo which contain high proportions of chloroplasts (Alquézar et al., 2009; Mendes et al., 2011; Zhang et al., 2012b). This study investigated the expression patterns of *CsLCYb1* promoter by stable genetic transformation in *Arabidopsis*. The results showed that promoter activity was highly correlated with seedling development and that GUS staining was observed clearly in leaf tissues, while little or no GUS staining was observed in other tissues (fruits, flowers, stems, and roots; **Figure 4**). A similar result was observed in transgenic *Arabidopsis* expressing the citrus *PSY* promoter-*GUS* construct (Zeng et al., 2013). These findings indicate that the *CsLCYb1* promoter is developmentally and tissue-specifically regulated. The expression patterns of *CsLCYb1* promoter determined by *GUS* expression were similar to the endogenous gene expression profiles in citrus, confirming that the *CsLCYb1* promoter is strictly regulated.

As secondary metabolites, carotenoids play vital roles in plant stress resistance. Previous studies have revealed that when plants suffer from environmental stresses, the expression of the genes for carotenoid biosynthesis will increase to produce more antioxidative components for enhancing plant resistance (Giuliano et al., 1993; Fanciullino et al., 2014). In addition to environmental cues, the elegant modulation of carotenoid metabolism is also tightly coordinated by endogenous signals, such as hormone levels. The hormonal regulation network of carotenoid metabolism has been reviewed previously (Osorio et al., 2013; Liu et al., 2015). This study investigated the responses of different *CsLCYb1* promoter fragments to various stimuli in transgenic citrus callus. The results showed that the promoter activity of *CsLCYb1* was induced by most stimuli, such as GA, IAA, and Glu (**Figure 5**). Some results are consistent with the change of *CsLCYb1* gene expression under the same treatments (Zhang et al., 2012a; Su et al., 2015). Zeng et al. (2013) also reported that the promoter activity of citrus *PSY* (the key rate-limiting enzyme in the carotenoid biosynthetic pathway) was affected by various stimuli. Additionally, we found that the *GUS* expression levels driven by different *CsLCYb1* promoter deletions were influenced by NaCl treatment (**Figure 5**), suggesting that the promoter activity of *CsLCYb1* could respond to salt stress. Very weak GUS staining was observed at the mechanical cut ends of some transgenic *Arabidopsis* tissues (**Figure 4**), indicating that the promoter activity of *CsLCYb1* may be induced by oxidative stress resulting from mechanical wound. Similar results were observed by Bouvier et al. (1998), who reported that the promoter activity of the *CCS* (which also has the function of lycopene β-cyclase) gene was dramatically activated by various ROS progenitors under different oxidative stress conditions. The stress-related and hormone-responsive *cis*-elements predicted in the promoter sequences (Supplementary Table S2) may partially explain the response of *CsLCYb1* promoter to various stimuli. However, this explanation remains to be further verified. *LCYb1* is highly expressed in green tissues, and is involved in plant photosynthesis and photoprotection. Previous studies reported that the mRNA transcripts of *LCYb1* in citrus are largely enhanced by light (Gao et al., 2011; Ma et al., 2012, 2015). This study predicted many light-responsive elements in the promoter of *CsLCYb1* (Supplementary Table S2), suggesting that the promoter activity of *CsLCYb1* is likely affected by light. Overall, the above analyses illustrate that *CsLCYb1* promoter responds to various exogenous and endogenous factors, and that the regulation of this promoter is a complex process.

### Identification of a Novel Enhancer Element Conferring Strong Promoter Activity

Promoter deletion analyses performed in three types of transgenic species all demonstrated that a deletion from LP3 to LP4 resulted in a significant reduction of promoter activity. Finer deletion analysis revealed that a 20 bp fragment existing as a tandem repeat in the region between LP3 and LP4 is an enhancer element conferring strong promoter activity to the minimal promoter, since the reduced copy number of the 20 bp fragment in the full-length promoter resulted in considerable decrease of *GUS* expression (**Figure 6**). A similar finding was previously reported, which suggested that four tandem repeats of a 20 bp sequence in the promoter of the melon cucumisin gene are sufficient to confer fruit-specific gene expression pattern to the minimal promoter, and that the 20 bp sequence contains a regulatory enhancer (Yamagata et al., 2002). Bustos et al. (2010) reported that the fusion of four tandem copies of a P1BS element (PHOSPHATE STARVATION RESPONSE REGULATOR 1, PHR1 binding sequences) to a 35S minimal promoter is sufficient to confer Pi inducibility to the reporter gene. In the future work, we will fuse the enhancer element to the upstream of a 35S minimum promoter to observe whether the enhancer element activates the 35S minimum promoter activity.

*In silico* analysis of the 20 bp sequence identified several interesting *cis*-elements (Inr-element, GT-element, GT-1 motif, and GA-motif, etc.). Previous studies have reported that Inr-elements and GT-elements are present in the promoter of many light-regulated genes, and the GT-1 motifs are present in the promoter of stress-induced genes (Zhou, 1999; Nakamura et al., 2002; Park et al., 2004). These results further indicate that the novel enhancer element may respond to light and stresses. The GA-motif was also found in the promoter of *G. lutea* lycopene β-cyclase gene (JQ417648), suggesting a common regulatory mechanism. Additionally, the deletion of the 20 bp fragment may disrupt adjacent *cis*-elements, such as the ARR (*Arabidopsis* response regulator) transcription factor binding site (NGATT) existing in the enhancer region as four copies. The ARR proteins belong to the GARP superfamily, two members of which have recently been reported to be related to carotenogenesis. One member is the *GOLDEN2-LIKE* (*GLK*) gene, which controls the dominant Uniform ripening (U) locus of tomato fruit. Tomato carrying the u mutation produced fruit with defective chloroplasts and low levels of sugar and lycopene (Powell et al., 2012). The other member is the *ARABIDOPSIS PSEUDO RESPONSR REGULATOR2-like* (*APRR2-like*) gene, which affects plastid number and size in tomato fruit, and enhances the levels of chlorophyll in immature fruit and carotenoids in red ripe fruit (Pan et al., 2013). Welsch et al. (2003, 2007) identified an enhancer element ATCTA in the phytoene synthase promoter from *Arabidopsis* and further discovered that the transcription factor *RAP2.2* (a member of the APETALA2 (AP2)/ethylene-responsive element-binding protein) interacting with the SINAT2 (SEVEN IN ABSENTIA OF ARABIDOPSIS2, a RING finger protein) bound to the ATCTA element to coordinately regulate *AtPSY* expression. These analyses indicate that the enhancer element identified in this study could be used as a good candidate to seek upstream *trans*-acting factors.

### Differences in Copy Number of Enhancer Element in *LCYb1* Promoters from Different Citrus Species

Previous studies of the *ODORANT1* (*ODO1*) gene, a key regulator in the volatile benzenoid pathway in petals, identified an enhancer region which distinguished a fragrant from a non-fragrant petunias cultivar (Van Moerkercke et al., 2011). This study also investigated the sequence characteristics of *LCYb1* promoter from different citrus species, which have different carotenoid contents and compositions. The results showed that the copy numbers of the 20 bp enhancer element were different among these species (**Figure 7A**). These differences may affect the expression level of *LCYb1* gene, thus resulting in carotenoid diversity among different citrus species. However, this speculation needs to be confirmed by more experiments. Sweet orange is a natural hybrid of pummelo and mandarin (Xu et al., 2013). In sweet orange, we only successfully isolated one *LCYb1* promoter sequence, which is relatively more similar with the promoter from pummelo. The failure to clone the promoter of the other allele originating from mandarin may be due to the insertion of a large fragment of a *CTV* resistance gene. CTV is one of the most severe diseases of citrus in the world (Bar-Joseph et al., 1989). Previous studies have reported that most of mandarin species are relatively more resistant to certain CTV strains compared with pummelo and grapefruit (Garnsey et al., 1987; Moreno et al., 2008). This study discovered the insertion of a CTV resistance gene fragment only in mandarin, while not in pummelo and grapefruit. These findings suggest that the fragment insertion may be associated with the differences in CTV resistance among citrus species, which deserves further exploration. Due to the sexual compatibility between species, high frequency of bud mutations, long history of cultivation and wide dispersion, the taxonomy and phylogeny of citrus are still complicated, controversial and confusing (Nicolosi et al., 2000; Garcia-Lor et al., 2013). The sequence polymorphisms identified in the *LCYb1* promoters may provide potential molecular markers to investigate the diversity and relationship among citrus species. Indeed, a derived SSR marker based on the copy numbers of the 20 bp enhancer element in *LCYb1* promoters could distinguish mandarin, pummelo, grapefruit and orange species as shown by the study of 14 citrus species (**Figure 7B**), which provides an ideal molecular marker to study the genetic relationship between different species.

## Conclusion

This study elucidates the expression characteristics of the *LCYb1* promoter from citrus, thus facilitating the understanding of the complex regulatory mechanisms of *LCYb1* expression in higher plants. The identified novel enhancer element is required for promoter activity; besides, it also can be used as a marker to distinguish different citrus species. These data give a clue to further study of the differences in gene expression among species. The promoter cloned and functionally validated in this study would be an ideal candidate for genetic engineering and seeking of upstream *trans*-acting elements.

## Author Contributions

SL performed the major experiments and wrote the manuscript. YZ and KZ, assisted in the experiments. XZ give suggestions for writing improvement. QX designed the experiment. XD proposed and supervised the research, and provided valuable comments on the manuscript. All authors read and approved the manuscript.

## Conflict of Interest Statement

The authors declare that the research was conducted in the absence of any commercial or financial relationships that could be construed as a potential conflict of interest.

## Footnotes

1 http://citrus.hzau.edu.cn/orange/

2 http://linux1.softberry.com/

3 http://www.dna.affrc.go.jp/PLACE/signalscan.html

4 http://bioinformatics.psb.ugent.be/webtools/plantcare/html

5 https://phytozome.jgi.doe.gov/pz/portal.html#!info?aliasOrg_Cclementina
