# Supplementary material for: Isolation and Functional Characterization of a Lycopene β-cyclase Gene Promoter from Citrus
Source: Front Plant Sci. 2016 Sep 13;7:1367. doi: 10.3389/fpls.2016.01367 (PMC5020073; doi:10.3389/fpls.2016.01367)
Supplement: Supplementary file 1 [file Data_Sheet_1.DOCX]

**>pCsLCYb1**

AAACTTACCTGTTGCCTCTAATTTATCTTGAACTCGAATACATGTGCCATGTGGGTGACCCACTCGGGGTGGGTATGGCCTGAAATGCGTTTTGGTGGGTCACTATCACTTACTTTTTTATGGCTGACGTTCGCTCATCTCCCTCATCCCAAAAATATTATTTTTATTCTTATTTGGGTTATTTTTCGCCACCCCCATAAGAATGGACAAATTCTGTCCACTCACTTCTTTCAACAAATATTACAACGCACCCACTTTGTTTAGATTACCGTTAACTTTAACAATTTTTTAGTTATTATTTGAATTAATTTGGCTGCAAGTACTAAAATTACGCATCATAACTCAAACGAATTTAAAAAATGAAATATATCGTTATAATAAATATGATTTATTTTTATTTTTGTACTAATTTACTAATATAATTAAAGAAATATGTATAACATTGCTATAAAAACAAAAATTAAACTCGAAGGTATTAGAATTTTTACTTTCACATGTACTTGTAGTTTGTGTAGAACTTTTAATGATTAGTTAATCTTGAATCAGCAGGATGAATACCTAAAAAGGCATTCTAATACTCAAGAAAGCGCAACAATAACAACAATTGGAACTTCAATTTTTGTGCACAATTAAAATAAATATATTCCAAATAATTCACTCAAATCTAAAAACAGTAAACAATAACAAGCAGACTAAAAAAACATTAGGACTTTGATTTGCAGCCGCCAATCTCTAAGGCCGCGTCTGCTAGTAATAGCTTACCAAAGCATGCTTCTTATTGTATTGCAAAATCGGACTTCAATTTTACAACCATTAGTCTTTGACACATCTACTAGCAATAGCCCAAACCAATGTCTCACTTACGCCACTTGTTGAACTATAAAATCATATTTTTGGTGGTTGCATTTGCGACAGTTGGACCACTAGCTTCGTGACCGTTGTGACTGAAATCATCAACCCTTGATGAACATCCTTTGCTATTGGGCATGAATGGAGAAGGAAGAAAATGAGATTGAAGGAAGAAAAATGAGATTGAAGGAAGAAAAATGAGATTGAAGGAAGAAAAATGAGCGTGAAGGAGGAAAAGTGAGAAGAAAAAAAATTATATATTTTTTAATTATCCATAACTTATATTTACTATTCTACCCTTGTGACTGTGAAGTTATTGGTAATACTTCACAGATGGGTGTACGACGATAGTTGTTGAAAGAAGTGGGTGTGAGGTAAAATTTGTTGATTCTTATGGGGGTGTGGCGAAAAATAACCCTTTTTATTATTCACAAAATTCAGATTATTTAATTTCTCTTTCATTTATCGGATTATTCTTTTAATTTGTTCAGTTTGTCGTTGAGGACAGGCCACAAACGCAACACAAGCTTCATCTTTACCAAATTTCCGTAAGCAACTTCTGGGCTGAAAAATGCTCCCATTTCTCTCCTCTCTGCTTAATGGTAAGTCATCACATCTCTCTTTGCAATAGATTGAACAATTATTCCCTGAATTGATTCCTCTGTTTATAACTTCAACAAGACCCATATTCATTTCGTATTTCAAGGAGTCACGGATAACCCTTGTAGGAAAGCC

**>pCgLCYb1a**

AAACTTACCTGTTGCCTCTAATTTATCTTGAACTCGAATACATGTGCCATGTGGGTGACCCACTCGGGGTGGGTATGGCCTGAAATGCGTTTTGGTGGGTCACTATCACTTACTTTTTTATGGCTGACGTTCGCTCATCTCCCTCATCCCAAAAATATTATTTTTATTCTTATTTGGGTTATTTTTCGCTACCCCCATAAGAATGGACAAATTCTGTCCACTCACTTCTTTCAACAAATATTACAACGCACCCACTTTGTTAAGATTACCGTTAACTTTAACAATTTTTTAGTTATTATTTGAATTAATTTGGCTGCAAGTACTAAAATTACGCATCATAACTCAAACGAATTTAAAAAATGAAATATATCGTTATAACAAATATGATTTATTTTTATTTTTGTACTAATTTACTAATATAACTAAAGAAATATGTATAACATTGCTATAAAAACAAAAATTAAACTCGAAGGTATTAGAATTTTTACTTTCACATGTACTTGTAGTTTGTGTAGAACTTTTAATGATTAGTTAATCTTGAATCAGCAGGATGAATACCTAAAAAGGCACTCTAATACTCAAGAAAGCACAACAATAACAACAATTGGAACTTCAATTTTTGTGCACAATTAAAATAAATATATTCCAAATAATTCACTCAAATCTAAAAACAGTAAACAATAACAAGCAAACTAAAAAAACATTAGGACTTTGATTTGCAGCTGCCAATCTCTAAGGCCGCGTCTGCTAGTAATAGCTTACCAAAGCATGCTTCTTATTGTATTGCAAAATCGGACTTCAATTTTACAACCATTAGTCTCTGACACATCTACTAGTAATAGCCTAAACCAATGTCTCACTTACGCCTCTTGTTGAACTATAAAATCATACTTTTGGTGGTTGCATTTGCGACAGCTAAACCACTAGTTTCGTGACCGTTGTGATTGAAATCATCAACCCTTTATGAACATCCTTTGCTATTGGGCATGAATGGAGAAGGAAGAAAATGAGATTGAAGGAAGAAAAATGAGTGTGAAGGAGGAAAAGTGAGAAGAAAAAAAATTATATATTTTTTAATTATCCATAACTTATATTTACTATTCTACCCTTGTGACTGTGAAGTTATTGGTAATACTTCACAGATGGGTGTACGACGATAGTTGCTGAAAGAAGTGGGTGTGAGGTAAAATTTGTTGATTCTTATGGGGGTGTGGCGAAAAATAACCCTTTTATTATTCACAAAATTCAGATTATTTAATTTCTCTTTCATTTATCGGATTATTCTTTTAATTTGTTCAGTTTGTCGTTGAGGACAGGCCACAAACGCAACACAAGCTTCATCTTTACCAAATTTCCGTAAGCAACTTCTGGGCTGAAAAATGCTCCCATTTCTCTCCTCTCTGCTTAATGGTAAGTCATCACATCTCTCTTTGCAATAGATTGAACAATTATTCCCTGAATTGATTCCTCTGTTTATAACTTCAACAAGACCCATATTCATTTTGTATTTCAAGGAGTCACGGATAACCCTTGTAGGAAAGCC

**>pCgLCYb1b**

AAACTTACCTGTTGCCTCTAATTTATCTTGAACTCGAATACATGTGCCATGTGGGTGACCCACTCGGGGTGGGTATGGCCTGAAATGCGTTTTGGTGGGTCACTATCACTTACTTTTTTATGGCTGACGTTCGCTCATCTCCCTCATCCCAAAAATATTATTTTTATTCTTATTTGGGTTATTTTTCGCTACCCCCATAAGAATGGACAAATTCTGTCCACTCACTTCTTTCAACAAATATTACAACGCACCCACTTTGTTAAGATTACCGTTAACTTTAACAATTTTTTAGTTATTATTTGAATTAATTTGGCTGCAAGTACTAAAATTACGCATCATAACTCAAACGAATTTAAAAAATGAAATATATCGTTATAACAAATATGATTTATTTTTATTTTTGTACTAATTTACTAATATAATTAAAGAAATATGTATAACATTGCTATAAAAACAAAAATTAAACTCGAAGGTATTAGAATTTTTACTTTCACATGTACTTGTAGTTTGTGTAGAACTTTTAATGATTAGTTAATCTTGAATCAGCAGGATGAATACCTAAAAAGGCATTCTAATACTCAAGAAAGCGCAACAATAACAACAATTGGAACTTCAATTTTTGTGCACAATTAAAATAAATATATTCCAAATAATTCACTCAAATCTAAAAACAGTAAACAATAACAAGCAGACTAAAAAAACATTAGGACTTTGATTTGCAGCCGCCAATCTCTAAGGCCGCGTCTGCTAGTAATAGCTTACCGAAGCATGCTTCTTATTGTATTGCAAAATCGGACTTCAATTTTACAACCATTAGTCTCTGACACATCTACTAGCAATAGCCCAAACCAATGTCTCACTTACGCCACTTGTTGAACTATAAAATCATACTTTTGGTGGTTGCATTTGCGACAGTTGGACCACTAGCTTCGTGACCGTTGTGACTGAAATCATCAACCCTTGATGAACATCTTTTGCTATTGGGCATGAATGGAGAAGGAAGAAAATGAGATTGAAGGAAGAAAAATGAGATTGAAGGAGGAAAAATGAGCGTGAAGGAGGAAAAGTGAGAAGAAAAAAAATTATATATTTTTTAATTATCCATAACTTATATTTACTATTCTACCCTTGTGACTGTGAAGTTATTGGTAATACTTCACAGATGGGTGTACGACGATAGTTGTTGAAAGAAGTGGGTGTGAGGTAAAATTTGTTGATTCTTATGGGGGTGTGGCGAAAAATAACCCTTTTTATTATTCACAAAATTCAGATTATTTAATTTCTCTTTCATTTATCGGATTATTCTTTTAATTTGTTCAGTTTGTCGTTGAGGACAGGCCACAAACGCAACACAAGCTTCATCTTTACCAAATTTCCGTAAGCAACTTCTGGGCTGAAAAATGCTCCCATTTCTCTCCTCTCTGCTTAATGGTAAGTCATCACATCTCTCTTTGCAATAGATTGAACAATTATTCCCTGAATTGATTCCTCTGTTTATAACTTCAACAAGACCCATATTCATTTCGTATTTCAAGGAGTCACGGATAACCCTTGTAGGAAAGCC

**>pCpLCYb1a**

AAACTTACCTGTTGCCTCTAATTTATCTTGAACTCGAATACATGTGCCATGTGGGTGACCCACTCGGGGTGGGTATGGCCTGAAATGCGTTTTGGTGGGTCACTATCACTTACTTTTTTATGGCTGACGTTCGCTCATCTCCCTCATCCCAAAAATATTATTTTTATTCTTATTTGGGTTATTTTTCGCTACCCCCATAAGAATGGACAAATTCTGTCCACTCACTTCTTTCAACAAATATTACAACGCACCCACTTTGTTAAGATTACCGTTAACTTTAACAATTTTTTAGTTATTATTTGAATTAATTTGGCTGCAAGTACTAAAATTACGCATCATAACTCAAACGAATTTAAAAAATGAAATATATCGTTATAACAAATATGATTTATTTTTATTTTTGTACTAATTTACTAATATAACTAAAGAAATATGTATAACATTGCTATAAAAACAAAAATTAAACTCGAAGGTATTAGAATTTTTACTTTCACATGTACTTGTAGTTTGTGTAGAACTTTTAATGATTAGTTAATCTTGAATCAGCAGGATGAATACCTAAAAAGGCACTCTAATACTCAAGAAAGCACAACAATAACAACAATTGGAACTTCAATTTTTGTGCACAATTAAAATAAATATATTCCAAATAATTCACTCAAATCTAAAAACAGTAAACAATAACAAGCAAACTAAAAAAACATTAGGACTTTGATTTGCAGCTGCCAATCTCTAAGGCCGCGTCTGCTAGTAATAGCTTACCAAAGCATGCTTCTTATTGTATTGCAAAATCGGACTTCAATTTTACAACCATTAGTCTCTGACACATCTACTAGTAATAGCCTAAACCAATGTCTCACTTACGCCTCTTGTTGAACTATAAAATCATACTTTTGGTGGTTGCATTTGCGACAGCTAAACCACTAGTTTCGTGACCGTTGTGATTGAAATCATCAACCCTTTATGAACATCCTTTGCTATTGGGCATGAATGGAGAAGGAAGAAAATGAGATTGAAGGAAGAAAAATGAGTGTGAAGGAGGAAAAGTGAGAAGAAAAAAAATTATATATTTTTTAATTATCCATAACTTATATTTACTATTCTACCCTTGTGACTGTGAAGTTATTGGTAATACTTCACAGATGGGTGTACGACGATAGTTGCTGAAAGAAGTGGGTGTGAGGTAAAATTTGTTGATTCTTATGGGGGTGTGGCGAAAAATAACCCTTTTATTATTCACAAAATTCAGATTATTTAATTTCTCTTTCATTTATCGGATTATTCTTTTAATTTGTTCAGTTTGTCGTTGAGGACAGGCCACAAACGCAACACAAGCTTCATCTTTACCAAATTTCCGTAAGCAACTTCTGGGCTGAAAAATGCTCCCATTTCTCTCCTCTCTGCTTAATGGTAAGTCATCACATCTCTCTTTGCAATAGATTGAACAATTATTCCCTGAATTGATTCCTCTGTTTATAACTTCAACAAGACCCATATTCATTTTGTATTTCAAGGAGTCACGGATAACCCTTGTAGGAAAGCC

**>pCpLCYb1b**

AAACTTACCTGTTGCCTCTAATTTATCTTGAACTCGAATACATGTGCCATGTGGGTGACCCACTCGGGGTGGGTATGGCCTGAAATGCGTTTTGGTGGGTCACTATCACTTACTTTTTTATGGCTGACGTTCGCTCATCTCCCTCATCCCAAAAATATTATTTTTATTCTTATTTGGGTTATTTTTCGCTACCCCCATAAGAATGGACAAATTCTGTCCACTCACTTCTTTCAACAAATATTACAACGCACCCACTTTGTTAAGATTACCGTTAACTTTAACAATTTTTTAGTTATTATTTGAATTAATTTGGCTGCAAGTACTAAAATTACGCATCATAACTCAAACGAATTTAAAAAATGAAATATATCGTTATAACAAATATGATTTATTTTTATTTTTGTACTAATTTACTAATATAACTAAAGAAATATGTATAACATTGCTATAAAAACAAAAATTAAACTCGAAGGTATTAGAATTTTTACTTTCACATGTACTTGTAGTTTGTGTAGAACTTTTAATGATTAGTTAATCTTGAATCAGCAGGATGAATACCTAAAAAGGCACTCTAATACTCAAGAAAGCACAACAATAACAACAATTGGAACTTCAATTTTTGTGCACAATTAAAATAAATATATTCCAAATAATTCACTCAAATCTAAAAACAGTAAACAATAACAAGCAGACTAAAAAAACATTAGGACTTTGATTTGCAGCCGCCAATCTCTAAGGCCGCGTCTGCTAGTAATAGCTTACCAAAGCATGCTTCTTATTGTATTGCAAAATCGGACTTCAATTTTACAACCATTAGTCTTTGACACATCTACTAGCAATAGCCCAAACCAATGTCTCACTTACGCCACTTGTTGAACTATAAAATCATATTTTTGGTGGTTGCATTTGCGACAGTTGGACCACTAGCTTCGTGACCGTTGTGACTGAAATCATCAACCCTTGATGAACATCCTTTGCTATTGGGCATGAATGGAGAAGGAAGAAAATGAGATTGAAGGAAGAAAAATGAGATTGAAGGAAGAAAAATGAGATTGAAGGAAGAAAAATGAGCGTGAAGGAGGAAAAGTGAGAAGAAAAAAAATTATATATTTTTTAATTATCCATAACTTATATTTACTATTCTACCCTTGTGACTGTGAAGTTATTGGTAATACTTCACAGATGGGTGTACGACGATAGTTGCTGAAAGAAGTGGGTGTGAGGTAAAATTTGTTGATTCTTATGGGGGTGTGGCGAAAAATAACCCTTTTATTATTCACAAAATTCAGATTATTTAATTTCTCTTTCATTTATCGGATTATTCTTTTAATTTGTTCAGTTTGTCGTTGAGGACAGGCCACAAACGCAACACAAGCTTCATCTTTACCAAATTTCCGTAAGCAACTTCTGGGCTGAAAAATGCTCCCATTTCTCTCCTCTCTGCTTAATGGTAAGTCATCACATCTCTCTTTGCAATAGATTGAACAATTATTCCCTGAATTGATTCCTCTGTTTATAACTTCAACAAGACCCATATTCATTTCGTATTTCAAGGAGTCACGGATAACCCTTGTAGGAAAGCC

**>pCrLCYb1**

ACTTTGTTAAGATTACCGTTAACTTTAACAATTTTTTAGTTATTATTTGAATTAATTTGGCTGCAAGTACTAAAATTACGCATCATAACTCAAACGAATTTAAAAAATGAAATATATCGTTATAATAAATACGATTTATTTTTATTTTTGTACTAATTTACTAATATAATTAAAGAAATATGTATAACAGTGGTATAAAAACAAAAATCAAACTCGAAGGTATTACAATTTTTACTTTCCCATGTACTTGTAGTTTGTGTAGAACTTTTAATGATTAGTTAATCTTGAATCAGCAGGATGAATACCTAAAAAGGCACTCTAATACTCAAGAAAGCACAACAATAACAACAATTAGAACTTCAATTTTTGTGCACAATTAAAATAAATATATTCTAAATAATTCACTCAAATTTAAAAACGATAAACAATAACAAGCAGACTCAAAAAACATTAGGACTTTGATTTGCAGCCGCCAATCTCTAAAGCCGCGTTTGCTAGTAATAGCTCACCGAAGTGTGCTTCTTATTGCATTGCAAAATCGGACTTCAATTTTACAACCATCGGTCTTTGACACATCTTCTAGCAACATCCCAAACCAATGTCTCACTTACGCCTCTTGTTGAACTATAAAATTAGACTTTTGGTGGTTGCATTTGCGACAGTTGGACCACTAGCTTTGTGATCCTTGTGACTAAAATCATCAACCCTTGATGAATATCCTTCGCTATTGGGCATGAATGGAGACGGAAGAAAATGAGATTGAAGGAAGAAAAATGAGCGTGAGGGAGGAAATGCGAGAAGAAAAAAATTTTTTTTTTTTTAATTATCCATAATGATAAGTGCATATTTTCCATATATTTTGCTATTAATTTCTCTATCTTTTTCTTGTTTTGGTCTTAAATATTCTAGTTATTTTAGTTAATTTTTAATTTAGTAAATTATATTTTATTATGTTAATTTTATGTTAATTTTTATATTTTGTTATTTTAGGTGCATTTTGGGAATAAAGAGGAATTAAATCAGAATATTCAAGTAAGGAGAGCTTAAAAAAGAAACTAATTAGAAATTTAAAGAAAGTAATGGGAGTTAAAATGGAAGAAAATCAAAGCAACCAATGCCTAATTGAAGTTCATACGTGAAGATGAGAAGAATTAGCATTGGAAATAATTTAATTAATAAGGCAAACACTTCTTGTGATGGGCTTTGGCCAAATTGTTATTGGCTTATTAATTAAATAAGTCACGTGCATGGCATTGAATTATGGAAAGCAAAGTGAAACAAAAAGGGAAAATGGCCTTGCACGTGAATCCAAAGAAAAGTGGGGGCTTGACTTTTGTCTTTAATCCTTTTCATGTGAGGATTTAAGGCACAATATAAAAGAGGGCAGATGCAAGGAAAAGAAAAAAAAAAAACCAACAGCTGCTGGATGGAAACAGAGGAGGGCAGACGCAATTGGAGCAAGAGAATTTGAGATTGCAGCCGCAAGAAGGAAAAAAAAAACAAGGAAGACGCCAGAAGGAATTCAATTGAAGAAAAAAGGCAGCCGTTTAAATTAATCTCCATGATTGGTTTTGTCAATTCTCTTATTCCCAATTCAATTATGTTAAGCTATATTTTTATTTGGTTAAGGGTGAATTCAAAACCCTAAATATGATATGCAATTAAATTTAAATTCTATCATTCAATTTATTTAATTGAGATTGTTTATGTTCTTCTATAATTAATGTTCATGGTTTATTCTTGTTTTATTTAAGTGGCCAATTAAATAGGACTTGAATAAATATTATTGCTAGATTAAAATTCTTGACCCGTAATTGTCTTGATATTTTAATCATTAGTAGCAGGTTGGAATTAGTGATTTCAATTGAAGAACATAACCTAGGTTAAATAATCCGAGCTTGTGTGTTTATTGCCTAGATCAACCTAATATCTTTCTTTATTTAATGCTTCCATTATCTTAAATTTAAAGAGCTTGTTTTAAATTATTTAATGGTTAGAGATTATGTGAAGAGCTTGTTTTGCCTAACAACACATTAAGGAAAGATTGATACTAACAGAGCTTATTGTTGTCTACGGTTGATAATTTTAATATAAATTGATAATAGAATTGATATATTATGTGCATGTTTAGTGGTGGTGAATGATCCTTTAACCAAAGTTTTCGTTATTATTATTTCTTTCTCCTTTAATTAGAGTTTTTTTTCTTGTTCGTAATTTTAATTTCTTTATTTCTTACTATTTAGTTAAAAATTCATAAACCCCCCTTTTTATTTTTAATTGGCATTTTCTTTAGTTAGATTAATTTATATTATTATTACTATTACTATTATTTTATTTTAAATCTTGCTTTGGAGTTAATAATAAATATAACTAGCATAGTCTCTGTGGGATCGATCATTACTCGCTCTGTACTAATTATTTATATTAGTGGTAAGGTTTTAAATTTGGTGCACCTTAACGACACTACCACATAACTTATATTTACTATTCAACCCTCGTGACTGTGAAGTTATTTGTAATACTTCATAGATGGGTGTACGACGATAGTTGTTGAAAGAAGTGGGTGTGAGATAAAATTTGTTGATTCTTATGGGGGTGTGGCGAAAAATAACCCTTTTTATTATTCACAAAATTCAGATTATTTAATTTCTCTTTCATTTGTCGGATTATTCTTTTAATTTGTTCAGTTTGTCGTGAGGACAGGCCACACACGCAACACAAGCTTCATCTTTACCAAATATCCGTAAGCAACTTCTGGGCTGAAAAATGCTCCCATTTCTCTCCTCTCTGCTTAATGGTAAGTCATCACATCTCTCTTTGCAATAGATTGAACAATTATTCCCTGAATTGACTCCTCTGTTTATAACTTCAACAAGACCCATATTCATATTGTATTTCAAGGAGTCACGGATAACCCTTGTAGGAAAGCC

Figure S1. *LCYb1* promoter sequences from four citrus varieties. pCsLCYb represents *LCYb1* promoter isolated from ‘Anliu’ sweet orange (*Citrus sinensis*). pCgLCYb1a and pCgLCYb1b represent two different *LCYb1* promoters isolated from White-flesh ‘Guanxi’ pummelo (*Citrus grandis*). pCpLCYb1a and pCpLCYb1b represent two different *LCYb1* promoters isolated from Star Ruby grapefruit (*Citrus paradisi*). pCrLCYb represents *LCYb1* promoter obtained from the Citrus clementine genome databases. All the promoters are located upstream from the ATG start codon. The 20 bp enhancer elements are underlined in red color. The primer sequences used for SSR analyses are double underlined in green color. The inserted large fragment in the *LCYb1* promoter of mandarin are shaded.
