# Supplementary material for: Isolation and Functional Characterization of a Lycopene β-cyclase Gene Promoter from Citrus
Source: Front Plant Sci. 2016 Sep 13;7:1367. doi: 10.3389/fpls.2016.01367 (PMC5020073; doi:10.3389/fpls.2016.01367)
Supplement: Supplementary file 2 [file Data_Sheet_2.DOCX]

* 20 * 40 * 60 * 80 * 10
pCgLCYb1a : AAACTTACCTGTTGCCTCTAATTTATCTTGAACTCGAATACATGTGCCATGTGGGTGACCCACTCGGGGTGGGTATGGCCTGAAATGCGTTTTGGTGGG : 1445
pCpLCYb1a : AAACTTACCTGTTGCCTCTAATTTATCTTGAACTCGAATACATGTGCCATGTGGGTGACCCACTCGGGGTGGGTATGGCCTGAAATGCGTTTTGGTGGG : 1445
pCgLCYb1b : AAACTTACCTGTTGCCTCTAATTTATCTTGAACTCGAATACATGTGCCATGTGGGTGACCCACTCGGGGTGGGTATGGCCTGAAATGCGTTTTGGTGGG : 1466
pCpLCYb1b : AAACTTACCTGTTGCCTCTAATTTATCTTGAACTCGAATACATGTGCCATGTGGGTGACCCACTCGGGGTGGGTATGGCCTGAAATGCGTTTTGGTGGG : 1485
pCsLCYb1 : AAACTTACCTGTTGCCTCTAATTTATCTTGAACTCGAATACATGTGCCATGTGGGTGACCCACTCGGGGTGGGTATGGCCTGAAATGCGTTTTGGTGGG : 1486

 0 * 120 * 140 * 160 * 180 * 2
pCgLCYb1a : TCACTATCACTTACTTTTTTATGGCTGACGTTCGCTCATCTCCCTCATCCCAAAAATATTATTTTTATTCTTATTTGGGTTATTTTTCGCTACCCCCAT : 1346
pCpLCYb1a : TCACTATCACTTACTTTTTTATGGCTGACGTTCGCTCATCTCCCTCATCCCAAAAATATTATTTTTATTCTTATTTGGGTTATTTTTCGCTACCCCCAT : 1346
pCgLCYb1b : TCACTATCACTTACTTTTTTATGGCTGACGTTCGCTCATCTCCCTCATCCCAAAAATATTATTTTTATTCTTATTTGGGTTATTTTTCGCTACCCCCAT : 1367
pCpLCYb1b : TCACTATCACTTACTTTTTTATGGCTGACGTTCGCTCATCTCCCTCATCCCAAAAATATTATTTTTATTCTTATTTGGGTTATTTTTCGCTACCCCCAT : 1386
pCsLCYb1 : TCACTATCACTTACTTTTTTATGGCTGACGTTCGCTCATCTCCCTCATCCCAAAAATATTATTTTTATTCTTATTTGGGTTATTTTTCGCCACCCCCAT : 1387

 00 * 220 * 240 * 260 * 280 *
pCgLCYb1a : AAGAATGGACAAATTCTGTCCACTCACTTCTTTCAACAAATATTACAACGCACCCACTTTGTTAAGATTACCGTTAACTTTAACAATTTTTTAGTTATT : 1247
pCpLCYb1a : AAGAATGGACAAATTCTGTCCACTCACTTCTTTCAACAAATATTACAACGCACCCACTTTGTTAAGATTACCGTTAACTTTAACAATTTTTTAGTTATT : 1247
pCgLCYb1b : AAGAATGGACAAATTCTGTCCACTCACTTCTTTCAACAAATATTACAACGCACCCACTTTGTTAAGATTACCGTTAACTTTAACAATTTTTTAGTTATT : 1268
pCpLCYb1b : AAGAATGGACAAATTCTGTCCACTCACTTCTTTCAACAAATATTACAACGCACCCACTTTGTTAAGATTACCGTTAACTTTAACAATTTTTTAGTTATT : 1287
pCsLCYb1 : AAGAATGGACAAATTCTGTCCACTCACTTCTTTCAACAAATATTACAACGCACCCACTTTGTTTAGATTACCGTTAACTTTAACAATTTTTTAGTTATT : 1288

 300 * 320 * 340 * 360 * 380 *
pCgLCYb1a : ATTTGAATTAATTTGGCTGCAAGTACTAAAATTACGCATCATAACTCAAACGAATTTAAAAAATGAAATATATCGTTATAACAAATATGATTTATTTTT : 1148
pCpLCYb1a : ATTTGAATTAATTTGGCTGCAAGTACTAAAATTACGCATCATAACTCAAACGAATTTAAAAAATGAAATATATCGTTATAACAAATATGATTTATTTTT : 1148
pCgLCYb1b : ATTTGAATTAATTTGGCTGCAAGTACTAAAATTACGCATCATAACTCAAACGAATTTAAAAAATGAAATATATCGTTATAACAAATATGATTTATTTTT : 1169
pCpLCYb1b : ATTTGAATTAATTTGGCTGCAAGTACTAAAATTACGCATCATAACTCAAACGAATTTAAAAAATGAAATATATCGTTATAACAAATATGATTTATTTTT : 1188
pCsLCYb1 : ATTTGAATTAATTTGGCTGCAAGTACTAAAATTACGCATCATAACTCAAACGAATTTAAAAAATGAAATATATCGTTATAATAAATATGATTTATTTTT : 1189

 400 * 420 * 440 * 460 * 480 *
pCgLCYb1a : ATTTTTGTACTAATTTACTAATATAACTAAAGAAATATGTATAACATTGCTATAAAAACAAAAATTAAACTCGAAGGTATTAGAATTTTTACTTTCACA : 1049
pCpLCYb1a : ATTTTTGTACTAATTTACTAATATAACTAAAGAAATATGTATAACATTGCTATAAAAACAAAAATTAAACTCGAAGGTATTAGAATTTTTACTTTCACA : 1049
pCgLCYb1b : ATTTTTGTACTAATTTACTAATATAATTAAAGAAATATGTATAACATTGCTATAAAAACAAAAATTAAACTCGAAGGTATTAGAATTTTTACTTTCACA : 1070
pCpLCYb1b : ATTTTTGTACTAATTTACTAATATAACTAAAGAAATATGTATAACATTGCTATAAAAACAAAAATTAAACTCGAAGGTATTAGAATTTTTACTTTCACA : 1089
pCsLCYb1 : ATTTTTGTACTAATTTACTAATATAATTAAAGAAATATGTATAACATTGCTATAAAAACAAAAATTAAACTCGAAGGTATTAGAATTTTTACTTTCACA : 1090

 500 * 520 * 540 * 560 * 580 *
pCgLCYb1a : TGTACTTGTAGTTTGTGTAGAACTTTTAATGATTAGTTAATCTTGAATCAGCAGGATGAATACCTAAAAAGGCACTCTAATACTCAAGAAAGCACAACA : 950
pCpLCYb1a : TGTACTTGTAGTTTGTGTAGAACTTTTAATGATTAGTTAATCTTGAATCAGCAGGATGAATACCTAAAAAGGCACTCTAATACTCAAGAAAGCACAACA : 950
pCgLCYb1b : TGTACTTGTAGTTTGTGTAGAACTTTTAATGATTAGTTAATCTTGAATCAGCAGGATGAATACCTAAAAAGGCATTCTAATACTCAAGAAAGCGCAACA : 971
pCpLCYb1b : TGTACTTGTAGTTTGTGTAGAACTTTTAATGATTAGTTAATCTTGAATCAGCAGGATGAATACCTAAAAAGGCACTCTAATACTCAAGAAAGCACAACA : 990
pCsLCYb1 : TGTACTTGTAGTTTGTGTAGAACTTTTAATGATTAGTTAATCTTGAATCAGCAGGATGAATACCTAAAAAGGCATTCTAATACTCAAGAAAGCGCAACA : 991

 600 * 620 * 640 * 660 * 680 *
pCgLCYb1a : ATAACAACAATTGGAACTTCAATTTTTGTGCACAATTAAAATAAATATATTCCAAATAATTCACTCAAATCTAAAAACAGTAAACAATAACAAGCAAAC : 851
pCpLCYb1a : ATAACAACAATTGGAACTTCAATTTTTGTGCACAATTAAAATAAATATATTCCAAATAATTCACTCAAATCTAAAAACAGTAAACAATAACAAGCAAAC : 851
pCgLCYb1b : ATAACAACAATTGGAACTTCAATTTTTGTGCACAATTAAAATAAATATATTCCAAATAATTCACTCAAATCTAAAAACAGTAAACAATAACAAGCAGAC : 672
pCpLCYb1b : ATAACAACAATTGGAACTTCAATTTTTGTGCACAATTAAAATAAATATATTCCAAATAATTCACTCAAATCTAAAAACAGTAAACAATAACAAGCAGAC : 891
pCsLCYb1 : ATAACAACAATTGGAACTTCAATTTTTGTGCACAATTAAAATAAATATATTCCAAATAATTCACTCAAATCTAAAAACAGTAAACAATAACAAGCAGAC : 892

 700 * 720 * 740 * 760 * 780 *
pCgLCYb1a : TAAAAAAACATTAGGACTTTGATTTGCAGCTGCCAATCTCTAAGGCCGCGTCTGCTAGTAATAGCTTACCAAAGCATGCTTCTTATTGTATTGCAAAAT : 752
pCpLCYb1a : TAAAAAAACATTAGGACTTTGATTTGCAGCTGCCAATCTCTAAGGCCGCGTCTGCTAGTAATAGCTTACCAAAGCATGCTTCTTATTGTATTGCAAAAT : 752
pCgLCYb1b : TAAAAAAACATTAGGACTTTGATTTGCAGCCGCCAATCTCTAAGGCCGCGTCTGCTAGTAATAGCTTACCGAAGCATGCTTCTTATTGTATTGCAAAAT : 773
pCpLCYb1b : TAAAAAAACATTAGGACTTTGATTTGCAGCCGCCAATCTCTAAGGCCGCGTCTGCTAGTAATAGCTTACCAAAGCATGCTTCTTATTGTATTGCAAAAT : 792
pCsLCYb1 : TAAAAAAACATTAGGACTTTGATTTGCAGCCGCCAATCTCTAAGGCCGCGTCTGCTAGTAATAGCTTACCAAAGCATGCTTCTTATTGTATTGCAAAAT : 793

 800 * 820 * 840 * 860 * 880 *
pCgLCYb1a : CGGACTTCAATTTTACAACCATTAGTCTCTGACACATCTACTAGTAATAGCCTAAACCAATGTCTCACTTACGCCTCTTGTTGAACTATAAAATCATAC : 653
pCpLCYb1a : CGGACTTCAATTTTACAACCATTAGTCTCTGACACATCTACTAGTAATAGCCTAAACCAATGTCTCACTTACGCCTCTTGTTGAACTATAAAATCATAC : 653
pCgLCYb1b : CGGACTTCAATTTTACAACCATTAGTCTCTGACACATCTACTAGCAATAGCCCAAACCAATGTCTCACTTACGCCACTTGTTGAACTATAAAATCATAC : 674
pCpLCYb1b : CGGACTTCAATTTTACAACCATTAGTCTTTGACACATCTACTAGCAATAGCCCAAACCAATGTCTCACTTACGCCACTTGTTGAACTATAAAATCATAT : 693
pCsLCYb1 : CGGACTTCAATTTTACAACCATTAGTCTTTGACACATCTACTAGCAATAGCCCAAACCAATGTCTCACTTACGCCACTTGTTGAACTATAAAATCATAT : 694

 900 * 920 * 940 * 960 * 980 *
pCgLCYb1a : TTTTGGTGGTTGCATTTGCGACAGCTAAACCACTAGTTTCGTGACCGTTGTGATTGAAATCATCAACCCTTTATGAACATCCTTTGCTATTGGGCATGA : 554
pCpLCYb1a : TTTTGGTGGTTGCATTTGCGACAGCTAAACCACTAGTTTCGTGACCGTTGTGATTGAAATCATCAACCCTTTATGAACATCCTTTGCTATTGGGCATGA : 554
pCgLCYb1b : TTTTGGTGGTTGCATTTGCGACAGTTGGACCACTAGCTTCGTGACCGTTGTGACTGAAATCATCAACCCTTGATGAACATCTTTTGCTATTGGGCATGA : 575
pCpLCYb1b : TTTTGGTGGTTGCATTTGCGACAGTTGGACCACTAGCTTCGTGACCGTTGTGACTGAAATCATCAACCCTTGATGAACATCCTTTGCTATTGGGCATGA : 594
pCsLCYb1 : TTTTGGTGGTTGCATTTGCGACAGTTGGACCACTAGCTTCGTGACCGTTGTGACTGAAATCATCAACCCTTGATGAACATCCTTTGCTATTGGGCATGA : 595


 1000 * 1020 * 1040 * 1060 * 1080
pCgLCYb1a : ATGGAGAAGGAAGAAAATGAGATTGAAGGAAGAAAAATGAG----------------------------------------TGTGAAGGAGGAAAAGTG : 495
pCpLCYb1a : ATGGAGAAGGAAGAAAATGAGATTGAAGGAAGAAAAATGAG----------------------------------------TGTGAAGGAGGAAAAGTG : 495
pCgLCYb1b : ATGGAGAAGGAAGAAAATGAGATTGAAGGAAGAAAAATGAGATTGAAGGAGGAAAAATGAG--------------------CGTGAAGGAGGAAAAGTG : 496
pCpLCYb1b : ATGGAGAAGGAAGAAAATGAGATTGAAGGAAGAAAAATGAGATTGAAGGAAGAAAAATGAGATTGAAGGAAGAAAAATGAGCGTGAAGGAGGAAAAGTG : 495
pCsLCYb1 : ATGGAGAAGGAAGAAAATGAGATTGAAGGAAGAAAAATGAGATTGAAGGAAGAAAAATGAGATTGAAGGAAGAAAAATGAGCGTGAAGGAGGAAAAGTG : 496

 **20bp enhancer element repeats**
 * 1100 * 1120 * 1140 * 1160 * 1180
pCgLCYb1a : AGAAGAAAAAAAATTATATATTTTTTAATTATCCATAACTTATATTTACTATTCTACCCTTGTGACTGTGAAGTTATTGGTAATACTTCACAGATGGGT : 396
pCpLCYb1a : AGAAGAAAAAAAATTATATATTTTTTAATTATCCATAACTTATATTTACTATTCTACCCTTGTGACTGTGAAGTTATTGGTAATACTTCACAGATGGGT : 396
pCgLCYb1b : AGAAGAAAAAAAATTATATATTTTTTAATTATCCATAACTTATATTTACTATTCTACCCTTGTGACTGTGAAGTTATTGGTAATACTTCACAGATGGGT : 397
pCpLCYb1b : AGAAGAAAAAAAATTATATATTTTTTAATTATCCATAACTTATATTTACTATTCTACCCTTGTGACTGTGAAGTTATTGGTAATACTTCACAGATGGGT : 396
pCsLCYb1 : AGAAGAAAAAAAATTATATATTTTTTAATTATCCATAACTTATATTTACTATTCTACCCTTGTGACTGTGAAGTTATTGGTAATACTTCACAGATGGGT : 397

 * 1200 * 1220 * 1240 * 1260 * 1280
pCgLCYb1a : GTACGACGATAGTTGCTGAAAGAAGTGGGTGTGAGGTAAAATTTGTTGATTCTTATGGGGGTGTGGCGAAAAATAACCCTTTT-ATTATTCACAAAATT : 298
pCpLCYb1a : GTACGACGATAGTTGCTGAAAGAAGTGGGTGTGAGGTAAAATTTGTTGATTCTTATGGGGGTGTGGCGAAAAATAACCCTTTT-ATTATTCACAAAATT : 298
pCgLCYb1b : GTACGACGATAGTTGTTGAAAGAAGTGGGTGTGAGGTAAAATTTGTTGATTCTTATGGGGGTGTGGCGAAAAATAACCCTTTTTATTATTCACAAAATT : 298
pCpLCYb1b : GTACGACGATAGTTGCTGAAAGAAGTGGGTGTGAGGTAAAATTTGTTGATTCTTATGGGGGTGTGGCGAAAAATAACCCTTTT-ATTATTCACAAAATT : 298
pCsLCYb1 : GTACGACGATAGTTGTTGAAAGAAGTGGGTGTGAGGTAAAATTTGTTGATTCTTATGGGGGTGTGGCGAAAAATAACCCTTTTTATTATTCACAAAATT : 298

 * 1300 * 1320 * 1340 * 1360 * 1380
pCgLCYb1a : CAGATTATTTAATTTCTCTTTCATTTATCGGATTATTCTTTTAATTTGTTCAGTTTGTCGTTGAGGACAGGCCACAAACGCAACACAAGCTTCATCTTT : 199
pCpLCYb1a : CAGATTATTTAATTTCTCTTTCATTTATCGGATTATTCTTTTAATTTGTTCAGTTTGTCGTTGAGGACAGGCCACAAACGCAACACAAGCTTCATCTTT : 199
pCgLCYb1b : CAGATTATTTAATTTCTCTTTCATTTATCGGATTATTCTTTTAATTTGTTCAGTTTGTCGTTGAGGACAGGCCACAAACGCAACACAAGCTTCATCTTT : 199
pCpLCYb1b : CAGATTATTTAATTTCTCTTTCATTTATCGGATTATTCTTTTAATTTGTTCAGTTTGTCGTTGAGGACAGGCCACAAACGCAACACAAGCTTCATCTTT : 199
pCsLCYb1 : CAGATTATTTAATTTCTCTTTCATTTATCGGATTATTCTTTTAATTTGTTCAGTTTGTCGTTGAGGACAGGCCACAAACGCAACACAAGCTTCATCTTT : 199

 * 1400 * 1420 * 1440 * 1460 * 1480
pCgLCYb1a : ACCAAATTTCCGTAAGCAACTTCTGGGCTGAAAAATGCTCCCATTTCTCTCCTCTCTGCTTAATGGTAAGTCATCACATCTCTCTTTGCAATAGATTGA : 100
pCpLCYb1a : ACCAAATTTCCGTAAGCAACTTCTGGGCTGAAAAATGCTCCCATTTCTCTCCTCTCTGCTTAATGGTAAGTCATCACATCTCTCTTTGCAATAGATTGA : 100
pCgLCYb1b : ACCAAATTTCCGTAAGCAACTTCTGGGCTGAAAAATGCTCCCATTTCTCTCCTCTCTGCTTAATGGTAAGTCATCACATCTCTCTTTGCAATAGATTGA : 100
pCpLCYb1b : ACCAAATTTCCGTAAGCAACTTCTGGGCTGAAAAATGCTCCCATTTCTCTCCTCTCTGCTTAATGGTAAGTCATCACATCTCTCTTTGCAATAGATTGA : 100
pCsLCYb1 : ACCAAATTTCCGTAAGCAACTTCTGGGCTGAAAAATGCTCCCATTTCTCTCCTCTCTGCTTAATGGTAAGTCATCACATCTCTCTTTGCAATAGATTGA : 100

 * 1500 * 1520 * 1540 * 1560 * 1580
pCgLCYb1a : ACAATTATTCCCTGAATTGATTCCTCTGTTTATAACTTCAACAAGACCCATATTCATTTTGTATTTCAAGGAGTCACGGATAACCCTTGTAGGAAAGCC : 1
pCpLCYb1a : ACAATTATTCCCTGAATTGATTCCTCTGTTTATAACTTCAACAAGACCCATATTCATTTTGTATTTCAAGGAGTCACGGATAACCCTTGTAGGAAAGCC : 1
pCgLCYb1b : ACAATTATTCCCTGAATTGATTCCTCTGTTTATAACTTCAACAAGACCCATATTCATTTCGTATTTCAAGGAGTCACGGATAACCCTTGTAGGAAAGCC : 1
pCpLCYb1b : ACAATTATTCCCTGAATTGATTCCTCTGTTTATAACTTCAACAAGACCCATATTCATTTCGTATTTCAAGGAGTCACGGATAACCCTTGTAGGAAAGCC : 1
pCsLCYb1 : ACAATTATTCCCTGAATTGATTCCTCTGTTTATAACTTCAACAAGACCCATATTCATTTCGTATTTCAAGGAGTCACGGATAACCCTTGTAGGAAAGCC : 1

Figure S2. Multiple sequence alignment of *LCYb1* promoters from sweet orange, pummelo, and grapefruit. The alignment is created by using ClustalX2 and Genedoc program. pCsLCYb1 represent *LCYb1* promoter from sweet orange (*Citrus sinensis*). pCgLCYb1a and pCgLCYb1b represent two different *LCYb1* promoters isolated from pummelo (*Citrus grandis*). pCpLCYb1a and pCpLCYb1b represent two different *LCYb1* promoters isolated from grapefruit (*Citrus paradisi*). Numbers indicate the positions relative to the ATG start codon (0). The same nucleotide acids in a given position are in white text on a black background. The different nucleotide acids in a given position are in black text on a white or gray background. The 20 bp enhancer element repeats are underlined and labeled.
