# Supplementary material for: Isolation and Functional Characterization of a Lycopene β-cyclase Gene Promoter from Citrus
Source: Front Plant Sci. 2016 Sep 13;7:1367. doi: 10.3389/fpls.2016.01367 (PMC5020073; doi:10.3389/fpls.2016.01367)
Supplement: Supplementary file 3 [file Data_Sheet_3.DOCX]

**Materials and Methods**

Real time PCR-based determination of transgene copy numbers in transgenic *Arabidopsis* was conducted according to the method described by [WENG et al. (2004](#_ENREF_1)). Two endogenous single-copy genes *DXR* (1-deoxy-D-xylulose 5-phosphate reductoisomerase) and *PDS* (Phytoene desaturation) in *Arabidopsis* were selected as reference genes. Briefly, genomic DNA was extracted from the leaves of transgenic *Arabidopsis* using the CTAB (cetyltrimethylammonium bromide) method (Cheng, 2003). DNA quantity and quality were checked using both the Nanodrop spectrophotometer and agarose gel electrophoresis. The oligonucleotide primers were designed with the Primer Express software (Applied Biosystems, Foster City, CA, USA) and listed below. All primers were synthesized at the Wuhan AuGCT Biotechnology Company (http://www.augct.com/).

The qRT-PCR analysis was carried out in 384-well plates with the ABI 7900 Real Time System (PE Applied Biosystems; Foster City, CA, USA) using KAPA ™ SYBR Green Master mixture (KAPA Biosystems Inc., Woburn, MA, USA). The reaction conditions were: 50℃ for 2 min, then 95℃ for 3 min, followed by 40 cycles at 95℃ for 3 s, 60℃ for 30 s. Fluorescent intensity data were collected at each annealing or extension step. Dissociation curves were used to validate the specificity of PCR reactions. For each biological sample, at least three technical replicates were performed. Output data were generated by the Sequence Detector version 1.3.1 software (Applied Biosystems, CA, USA) and exported to the Microsoft Excel program (Microsoft Office, 2010) for data analysis. The data were presented as mean ± SD of two independent experiments.

| **Table Primers for quantitative real-time PCR assay** | | | |
| --- | --- | --- | --- |
| Name | Sequence (5'-3') | length (bp) |  |
| DXR-RF | GAGTGTTTCACAGGTGGGTT | 97 |  |
| DXR-RR | GTTGTTGCTGCTGCACTTTC |  |  |
|  |  |  |  |
| PDS-RF | GAAATCAATGGGCCACCAAA | 100 |  |
| PDS-RR | CTTGGAAAATCCGACGAGCTT |  |  |
|  |  |  |  |
| GUS-RF | GCAGATGAACATGGCATCGT | 80 |  |
| GUS-RR | GCTTCGAAACCAATGCCTAAA |  |  |

**Results**

**(1) Dissociation curve**

***DXR*** **dissociation curve（Temperature 78.5℃）**


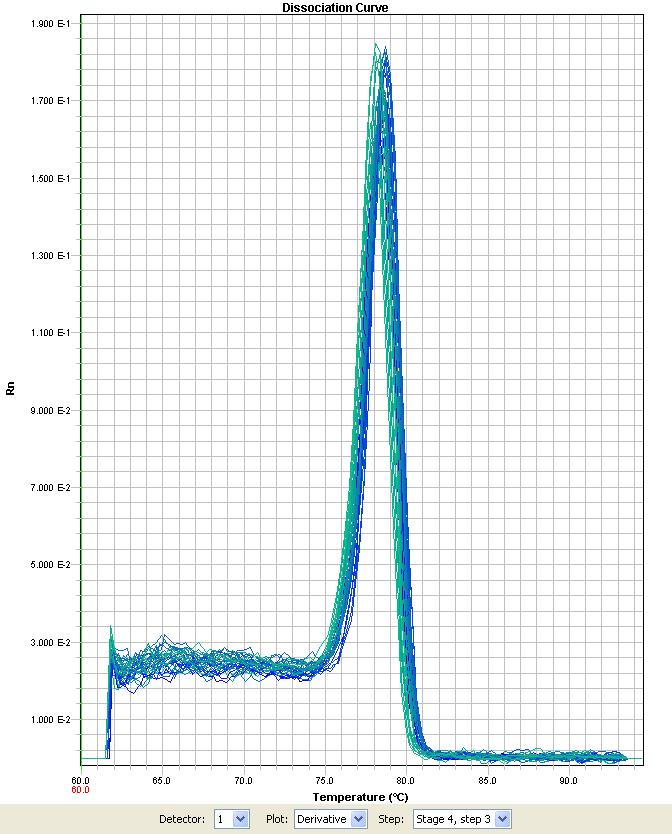


***PDS*** **dissociation curve（Temperature 83℃）**


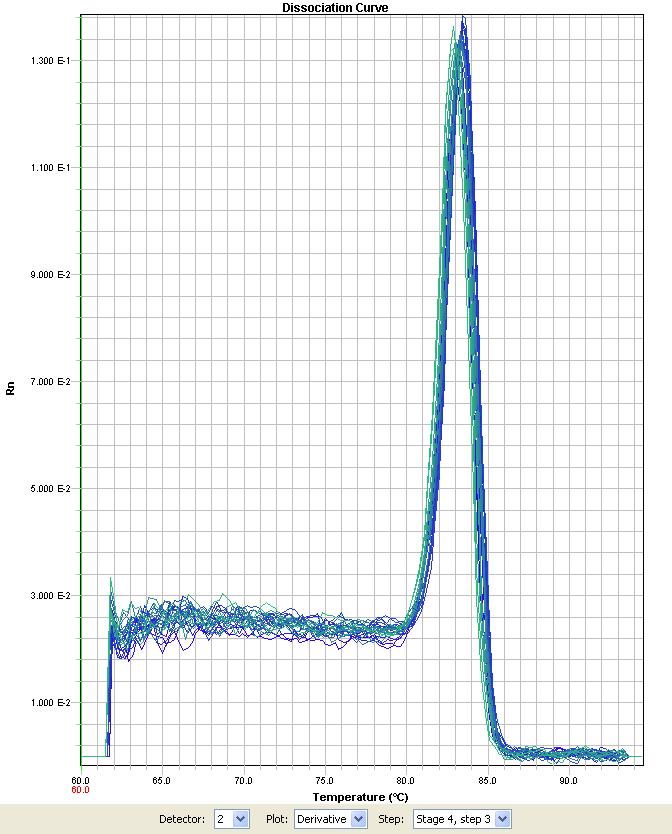


***GUS* dissociation curve（Temperature 79℃）**

**
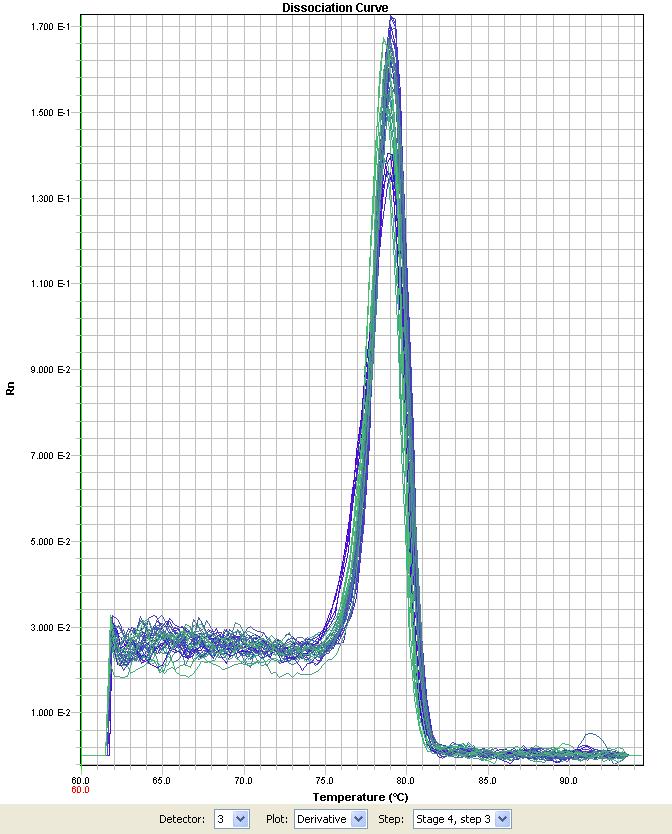
**

**(2) Standard amplification curves**

**The standard amplification curves of *DXR*, *PDS* and *GUS* were obtained by qRT-PCR assays of four serially diluted genomic DNA of the transgenic line 35S.**

***DXR* standard amplification curve; threshold, 0.197580**


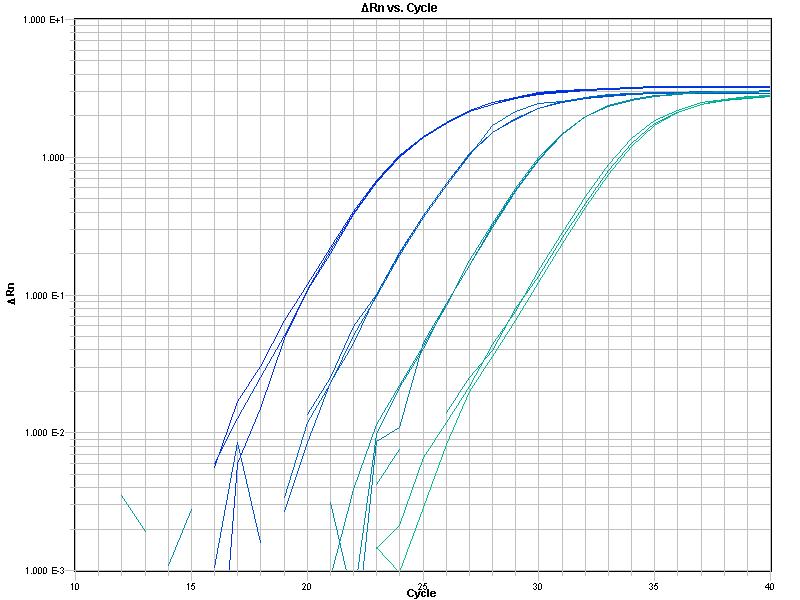


***PDS*** **standard amplification curve; threshold, 0.327623**


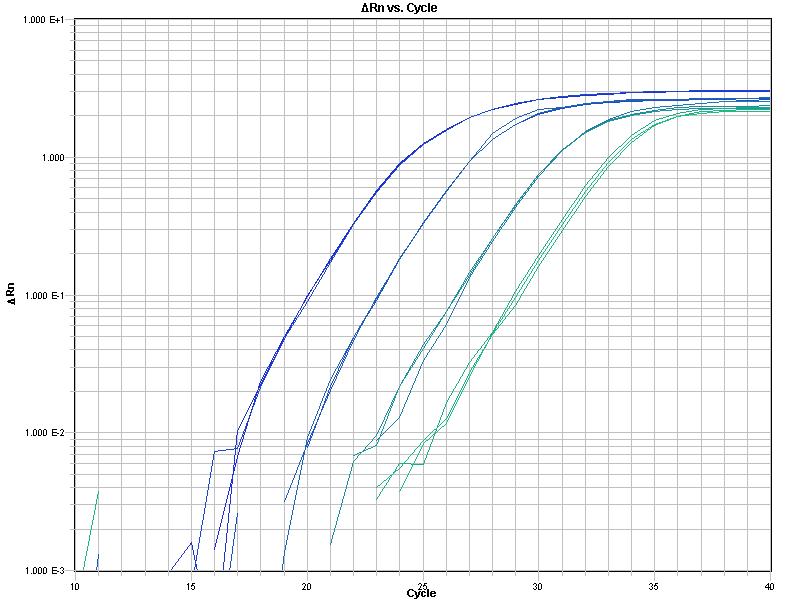


***GUS*** **standard amplification curve; threshold, 0.393999**


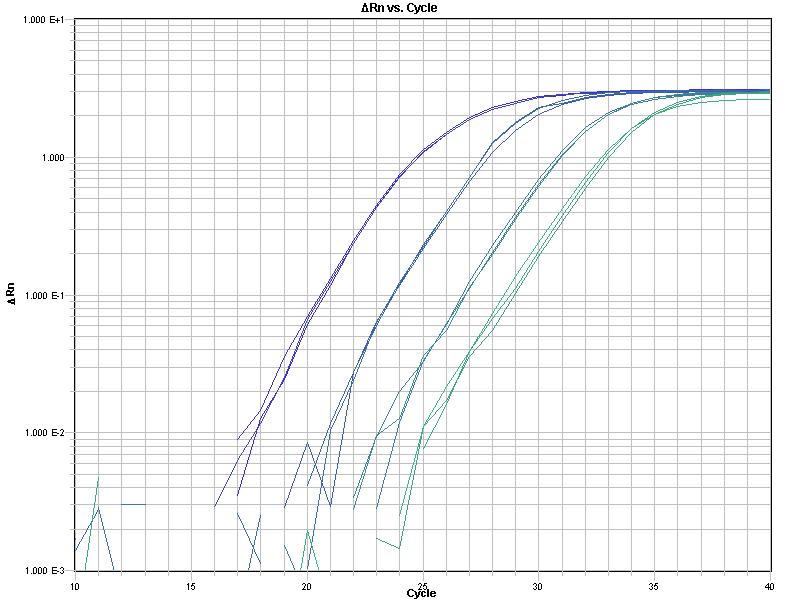


**(3) Amplification curves of three genes in different transgenic samples**

***DXR* amplification curves; threshold, 0.197580**


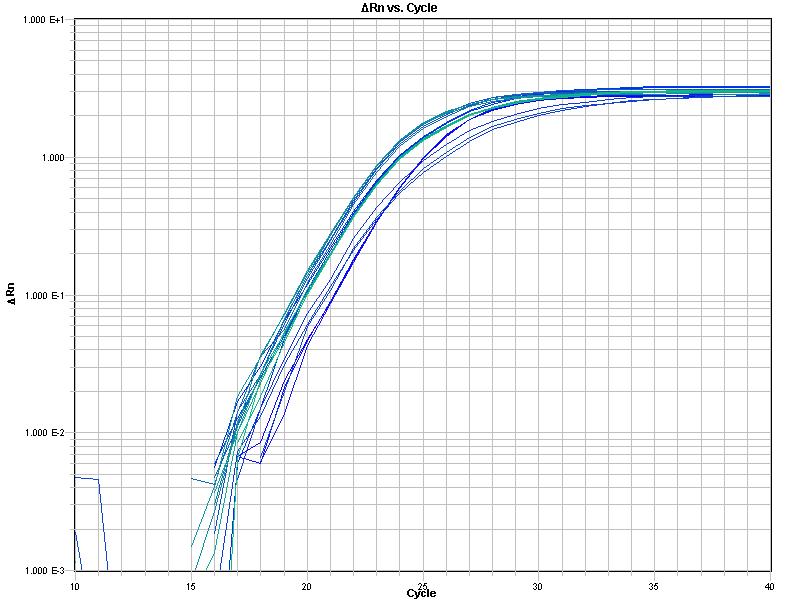


***PDS* amplification curves; threshold, 0.327623**


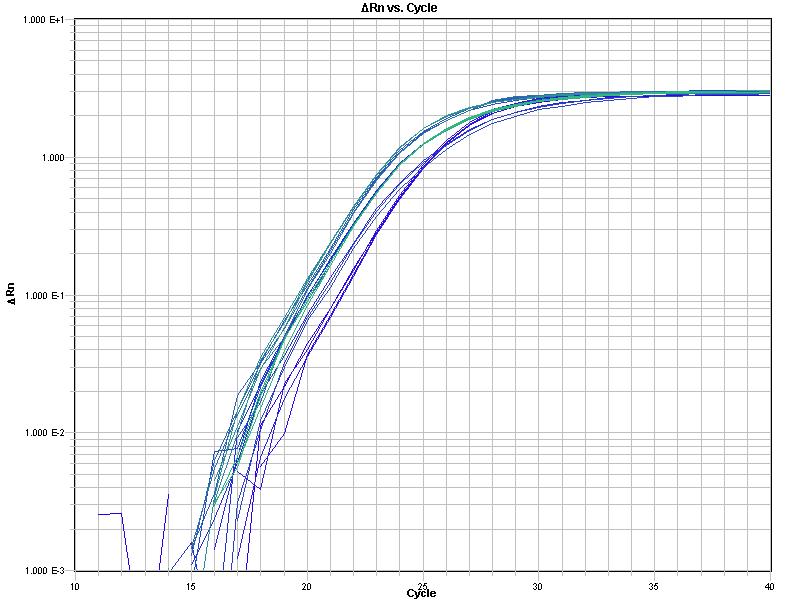


***GUS* amplification curves; threshold, 0.393999**


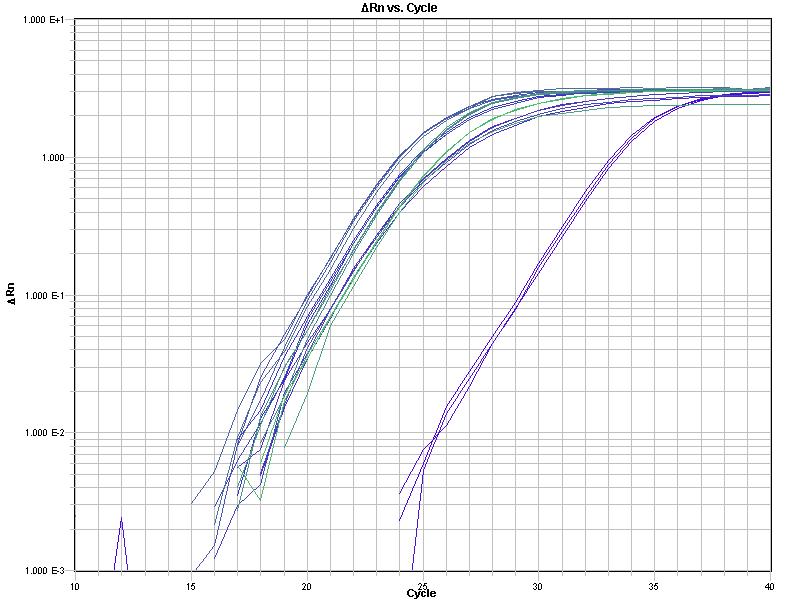


**(4) Ct values of three genes in the serially diluted transgenic sample 35S**

| Sample | DXR-Ct | PDS-Ct | GUS-Ct |
| --- | --- | --- | --- |
| **4** | 20.84±0.05 | 21.93±0.05 | 22.72±0.07 |
| **3** | 23.98±0.09 | 24.96±0.02 | 25.90±0.06 |
| **2** | 27.23±0.08 | 28.33±0.14 | 28.97±0.17 |
| **1** | 30.40±0.29 | 31.09±0.30 | 31.01±0.17 |
|  |  |  |  |

**(5) Ct values of three genes in each transgenic *Arabidopsis* sample**

| Sample | DXR-Ct | PDS-Ct | GUS-Ct |
| --- | --- | --- | --- |
| **WT** | 22.17±0.11 | 23.19±0.05 | 31.46±0.15 |
| **35S** | 21.62±0.25 | 22.66±0.17 | 23.73±0.10 |
| **LP** | 20.54±0.10 | 21.60±0.03 | 22.18±0.13 |
| **LP1** | 20.50±0.10 | 21.53±0.14 | 23.01±0.10 |
| **LP2** | 20.93±0.03 | 21.97±0.02 | 23.81±0.07 |
| **LP3** | 21.01±0.05 | 22.04±0.02 | 22.69±0.03 |
| **LP4** | 20.93±0.09 | 21.97±0.11 | 22.59±0.02 |
| **LP5** | 21.42±0.24 | 22.44±0.12 | 23.12±0.09 |
|  |  |  |  |

**(6) Standard equations of three genes**

***DXR* standard equation：Y = -3.1926 X + 33.596**

**（R^2^=1，suggesting a good correlation coefficient）**

***PDS*** **standard equation：Y = -3.0837 X + 34.286**

**（R^2^=0.9987，suggesting a good correlation coefficient）**

***GUS*** **standard equation：Y = -2.7929 X + 34.13**

**（R^2^=0.9906，suggesting a good correlation coefficient）**

**(7) Log10(CN) values in each sample calculated from the standard equations and the corresponding Ct values**

| Sample | DXR-Ct | PDS-Ct | GUS-Ct |
| --- | --- | --- | --- |
| **WT** | 3.58 | 3.60 | 0.96 |
| **35S** | 3.75 | 3.77 | 3.72 |
| **LP** | 4.09 | 4.11 | 4.28 |
| **LP1** | 4.10 | 4.14 | 3.98 |
| **LP2** | 3.97 | 3.99 | 3.69 |
| **LP3** | 3.94 | 3.97 | 4.09 |
| **LP4** | 3.97 | 3.99 | 4.13 |
| **LP5** | 3.81 | 3.84 | 3.94 |
|  |  |  |  |

**CN, Copy numbers**

**(8) Estimated copy numbers of *GUS* gene in each transgenic sample**

| Sample | GUS/DXR-CN | GUS/PDS-CN |
| --- | --- | --- |
| **WT** | 0.00 | 0.00 |
| **35S** | 0.94 | 0.90 |
| **LP** | 1.55 | 1.47 |
| **LP1** | 0.75 | 0.70 |
| **LP2** | 0.53 | 0.50 |
| **LP3** | 1.42 | 1.33 |
| **LP4** | 1.47 | 1.37 |
| **LP5** | 1.34 | 1.26 |
|  |  |  |

**These results showed that the copy numbers of *GUS* gene in each sample were 1 or 2, suggesting the single-copy insertion of the promoter-GUS fragment in transgenic *Arabidopsis*.**

WENG, HB., PAN, A., YANG, L., ZHANG, C., LIU, Z., ZHANG, D. (2004). Estimating Number of Transgene Copies in Transgenic Rapeseed by Real-Time PCR Assay With HMG I/Y as an Endogenous Reference Gene. Plant Mol Biol Rep. 22, 289-300. doi:
