# Supplementary figures and images for: Isolation and Functional Characterization of a Lycopene β-cyclase Gene Promoter from Citrus
Source: Front Plant Sci. 2016 Sep 13;7:1367. doi: 10.3389/fpls.2016.01367 (PMC5020073; doi:10.3389/fpls.2016.01367)

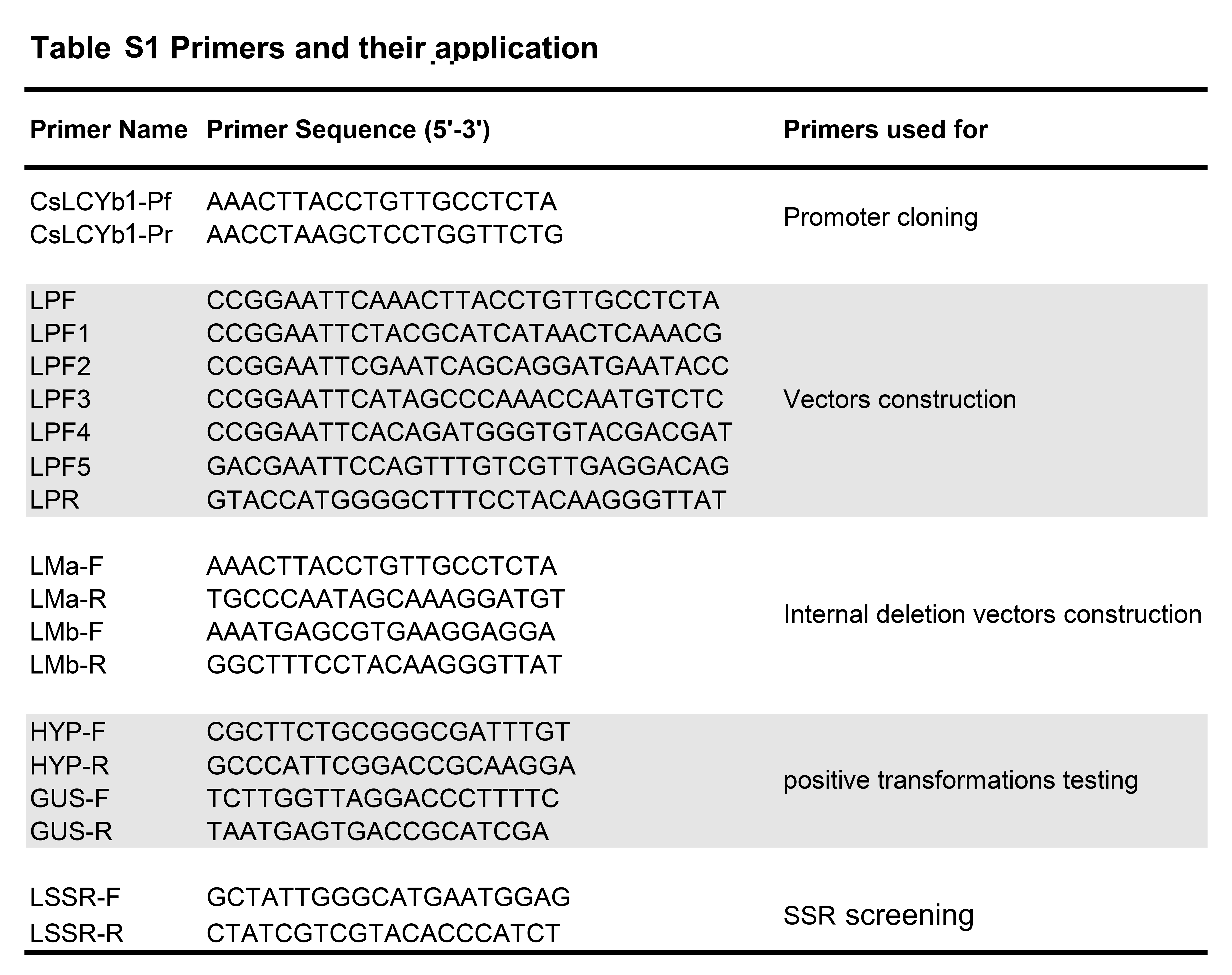

Supplement: Supplementary file 4 [file Image_1.TIF]

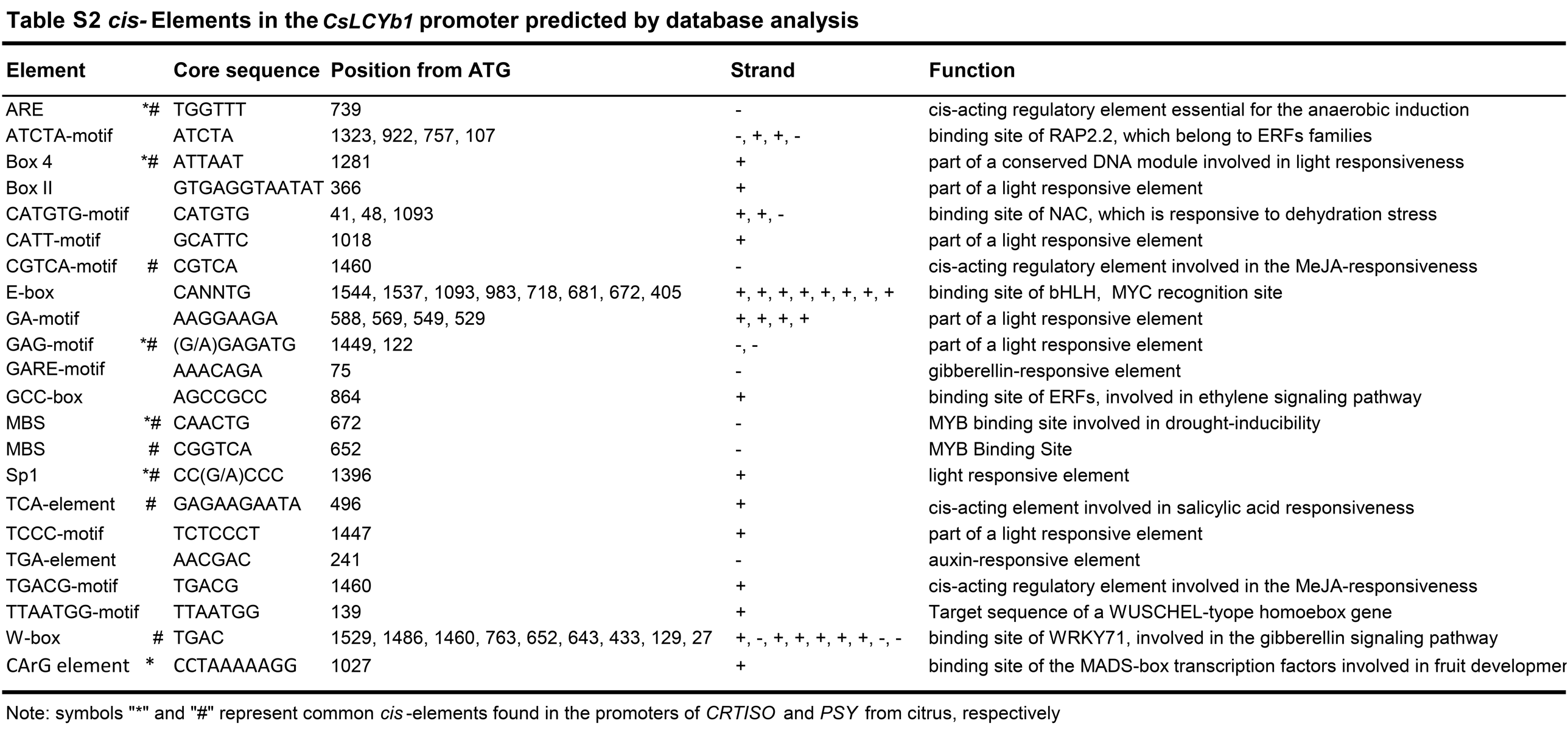

Supplement: Supplementary file 5 [file Image_2.TIF]
